# A pref-1-controlled non-inflammatory mechanism of insulin resistance

**DOI:** 10.1016/j.isci.2023.106923

**Published:** 2023-05-19

**Authors:** Yiheng Huang, Donghong Cui, Liujun Chen, Haibin Tong, Hong Wu, Grace K. Muller, Yadan Qi, Shuxia Wang, Jinjie Xu, Xiang Gao, Kathleen E. Fifield, Lingyan Wang, Zhengyuan Xia, Jacqueline L. Vanderluit, Suixin Liu, Lin Leng, Guang Sun, John McGuire, Lawrence H. Young, Richard Bucala, Dake Qi

**Affiliations:** 1 College of Pharmacy, Rady Faculty of Health Sciences, University of Manitoba, Winnipeg, MB, Canada; 2 Shanghai Key Laboratory of Psychotic Disorders, Shanghai Mental Health Center, Shanghai Jiao Tong University School of Medicine, Shanghai, China; 3 College of Life and Environment Sciences, Wenzhou University, Wenzhou, Zhejiang, China; 4 Institute of Cardiovascular Disease, Henan University of Chinese Medicine, Zhengzhou, Henan, China; 5 Department of Cell and Molecular Physiology, Loyola University, Chicago, IL, USA; 6 Department of Cardiology, The General Hospital of Chinese PLA, Beijing, China; 7 Beijing Anding Hospital, Capital Medical University, Beijing, China; 8 College of Life Sciences, Qingdao University, Qingdao, Shandong, China; 9 Division of Biomedical Sciences, Faculty of Medicine, Memorial University of Newfoundland, St. John’s, NL, Canada; 10 Guangdong Medical University, Zhanjiang, China; 11 Division of Cardiac Rehabilitation, Department of Physical Medicine & Rehabilitation, Xiangya Hospital of Central South University, Changsha, Hunan, China; 12 Department of Internal Medicine, Yale University School of Medicine, New Haven, CT, USA; 13 Department of Medical Biophysics, Schulich School of Medicine & Dentistry, Western University, London, ON, Canada; 14 Department of Cellular and Molecular Physiology, Yale University School of Medicine, New Haven, CT, USA

**Keywords:** Molecular biology, Immunology, Cell biology, Transcriptomics

## Abstract

While insulin resistance (IR) is associated with inflammation in white adipose tissue, we report a non-inflammatory adipose mechanism of high fat-induced IR mediated by loss of Pref-1. Pref-1, released from adipose Pref-1+ cells with characteristics of M2 macrophages, endothelial cells or progenitors, inhibits MIF release from both Pref-1+ cells and adipocytes by binding with integrin β1 and inhibiting the mobilization of p115. High palmitic acid induces PAR2 expression in Pref-1+ cells, downregulating Pref-1 expression and release in an AMPK-dependent manner. The loss of Pref-1 increases adipose MIF secretion contributing to non-inflammatory IR in obesity. Treatment with Pref-1 blunts the increase in circulating plasma MIF levels and subsequent IR induced by a high palmitic acid diet. Thus, high levels of fatty acids suppress Pref-1 expression and secretion, through increased activation of PAR2, resulting in an increase in MIF secretion and a non-inflammatory adipose mechanism of IR.

## Highlights

• Pref-1 inhibits MIF release from Pref-1+ cells and adipocytes in adipose tissue
• Pref-1 inhibits MIF by binding with integrin β1 and mediating p115 mobilization
• PAR2 downregulates Pref-1 from non-inflammatory cells in adipose tissue
• Reduction of Pref-1 in adipose tissue induces insulin resistance by increasing MIF

## Graphical abstract

**Figure:**
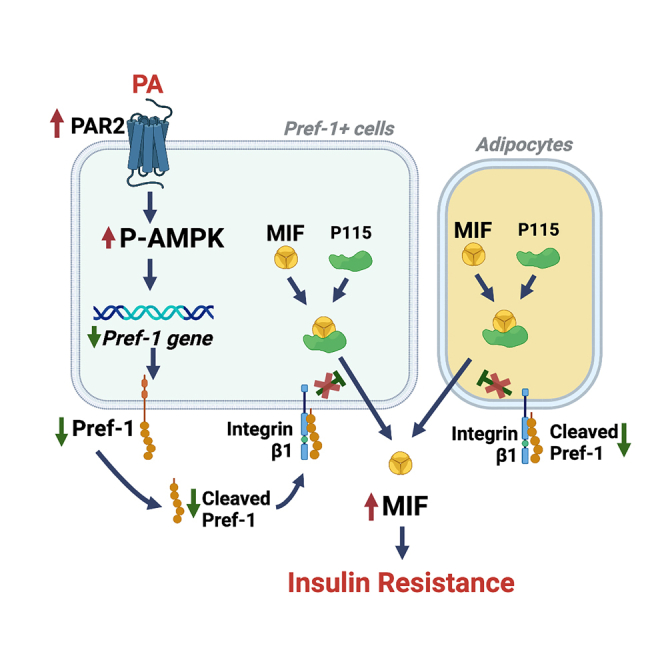

## Introduction

Insulin resistance (IR) in obesity is considered to arise from inflammation in white adipose tissue (WAT).^1^ However, anti-inflammatory therapies have failed to improve insulin sensitivity in animal models and human subjects.^2^^,^^3^ Recent studies further indicate that obesity-induced IR may occur without any change in systemic or tissue inflammation.^4^^,^^5^ IR can develop before macrophage accumulation and WAT inflammation,^5^ suggesting a proximate role of non-inflammatory adipose events in the initiation of IR. However, the underlying molecular and cellular mechanisms responsible for these observations remain largely unknown.

Macrophage migration inhibitory factor (MIF) is an evolutionarily conserved cytokine and upstream regulator of the innate immune response.^6^ However, MIF also has non-immune effects in the regulation of metabolic pathways and in myocardial stress and injury.^7^^,^^8^^,^^9^ Emerging work has shown that circulating MIF levels are significantly elevated in obesity, while weight loss reduces plasma MIF levels.^10^ *MIF* gene expression in abdominal fat, including visceral and subcutaneous adipose tissue is positively associated with waist circumference or body fat percentage in obesity.^11^^,^^12^ These findings suggest WAT as a potential source of circulating MIF.

In accord with previous paradigms regarding the pathogenesis of obesity, MIF expression in WAT is thought to arise from infiltrating macrophages.^13^^,^^14^ However, experimental findings also indicate that non-immune cells, such as progenitor adipocytes and adipocytes also release MIF under both physiologic and pathologic conditions,^15^^,^^16^ indicating an independent role for non-inflammatory mechanisms of adipose MIF production. At the current time, the pathways mediating non-inflammatory MIF release as well as their contribution to IR remain to be determined.

Preadipocyte factor-1 (Pref-1) is a transmembrane protein that is highly expressed in non-adipocyte cells in WAT. Pref-1 is cleaved by TNFα-converting enzyme to generate a soluble form, which acts as an autocrine/paracrine factor. Pref-1 was originally reported to regulate metabolism by inhibitingperoxisome proliferator activated receptor gamma (*PPARγ)* expression and adipogenesis.^17^ Mice with high levels of soluble Pref-1 in WAT have a reduced fat mass and hypertriglyceridemia.^18^ Mice lacking Pref-1 show augmented fat deposition and obesity.^19^ However, other investigators have reported that *pref-1* overexpression may improve glucose homeostasis during metabolic stress without changing adipogenesis.^20^ Thus, the details of the actions of the Pref-1 signaling pathway in regulating metabolism, especially in IR, have yet been clearly defined.

The present study investigates the hypothesis that reduced Pref-1 expression and release mediate IR by increasing MIF release from non-inflammatory cells in WAT. The collective findings, in both animal models and specimens from obese human subjects, reveal a functional interaction between Pref-1+ cells and adipocytes in WAT. Fatty acids and high fat diet reduce Pref-1 expression through activation of PAR2, with loss of Pref-1 leading to an increase in the extracellular release of MIF and plasma MIF levels.

## Results

### Pref-1 regulates non-inflammatory MIF release from WAT which induces IR

**Figure 1:**
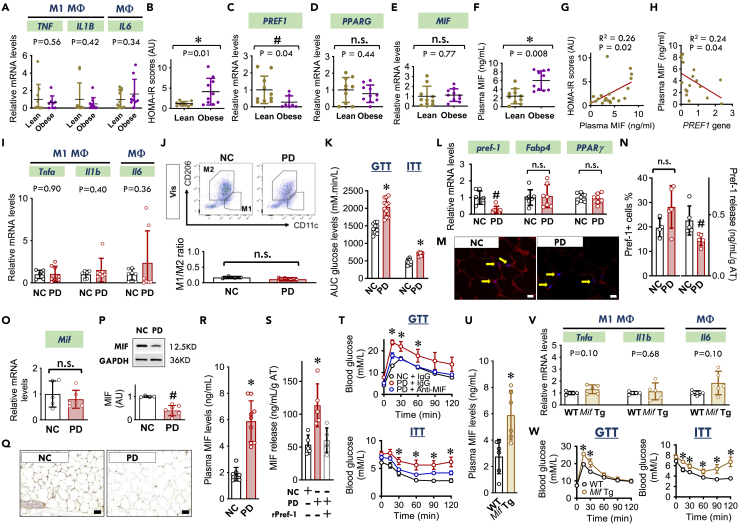
Pref-1 regulates non-inflammatory MIF release from WAT which induces IR (A) *TNF* (TNF-α), *IL1B* (IL-1β), and *IL6* (IL-6) mRNA expression in biopsied WAT from male subjects including lean (age: 23.3 ± 2.2; BMI ≤ 25 kg/m^2^) (N = 10) and obese individuals (age: 24.3 ± 3.3; BMI > 30 kg/m^2^) (N = 10). (B) HOMA insulin resistance scores (HOMA-IR scores) of recruited lean and obese individuals evaluated by plasma glucose and insulin levels. (C–F) *Pref. 1* (Pref-1) (C), *PPARG* (PPARγ) (D), and *MIF* (E) mRNA expression in biopsied WAT and plasma MIF levels (F) from recruited lean and obese individuals. (G and H) The correlation between HOMA-IR scores and plasma MIF levels, and the correlation between *Pref. 1* mRNA expression and plasma MIF levels. (I) Pro-inflammatory macrophage (M1 MΦ) markers: *Tnfa* (TNF-α) and *Il1b* (IL-1β), and general inflammatory (MΦ, macrophages) marker: *Il6* (IL-6) in visceral epididymal adipose tissue collected from WT mice (20-week-old) fed with normal chow (NC) or a high palmitic acid diet (PD) for 8 weeks. (J) The ratio of M1/M2 was evaluated by flow cytometry. (K) Insulin sensitivity was quantified by i.p. glucose tolerant test (GTT) and insulin tolerant test (ITT). (I and M) Adipose *pref-1*, *FABP4* and *PPARγ* mRNA expression (L) and Pref-1 immunofluorescence staining (M) from WT mice (20-week-old) fed with NC or PD for 8 weeks (Arrows, Pref-1 (red); Scale bars, 20 μm). (N) Pref-1+ cells sorted by flow cytometry from SVFs (left) and Pref-1 release from visceral adipose tissues (right) isolated from NC and PD groups. (O–Q) MIF gene and protein expression in WATs isolated from NC and PD groups (Scale bars, 20 μm). (R) Plasma MIF levels in NC and PD groups. (S) MIF release from WATs isolated from NC and PD groups in the presence or absence of recombinant Pref-1 proteins (2.5 μg/ml). (T) Insulin sensitivity quantified by GTT and ITT in PD groups following MIF neutralization with anti-MIF antibody (20 mg/kg, i.p. twice a week). (U) Plasma MIF levels in 25-week-old WT and *Mif* lung Tg mice. (V) Pro-inflammatory macrophage (M1 MΦ) markers: *Tnfa* (TNF-α) and *Il1b* (IL-1β), and general inflammatory (MΦ, macrophages) marker: *Il6* (IL-6) in visceral epididymal adipose tissue collected from 25-week-old WT and *Mif* lung Tg mice. (W) Insulin sensitivity quantified by GTT and ITT in 25-week-old WT and *Mif* lung Tg mice. All data are presented as mean ± SD. ^#^p ≤ 0.05 reduction vs. lean subjects in (C), vs. NC group in (N), and (P); ∗p ≤ 0.05 increase vs. lean subjects in (B) and (F), vs. NC group in (K) and (R), vs. WT group in (U) and (W), vs. other groups in (S) and (T). The n.s. represents no significance.

We sampled abdominal adipose tissue from lean (age: 23.3 ± 2.2; BMI < 25 kg/m^2^) and obese (age: 24.3 ± 3.3; BMI > 30 kg/m^2^) human subjects with metabolic dysfunction (Figures S1A–S1G). Subject-to-subject variability was detected within the recruited subjects, but the two groups nevertheless showed similar expression in adipose tissue of the inflammatory mediators: *TNF* (TNFα), *IL1B* (IL-1β), and *IL6* (IL-6) (Figure 1A). The obese group, however, had evidence of higher homeostatic model assessment for insulin resistance (HOMA IR) scores and lower adipose *Pref. 1* (Pref-1) gene expression (Figures 1B and 1C). The reduction in *Pref. 1* gene expression additionally was not accompanied by any change inperoxisome proliferator activated receptor gamma (*PPARG)* gene expression (Figure 1D). *MIF* gene expression in the sampled adipose was equivalent in both lean and obese groups (Figure 1E) and did not correlate with BMI (Figure S2). Nevertheless, the obese subjects had higher plasma MIF concentration (Figure 1F) that correlated positively with the subject’s IR (Figure 1G). More interestingly, adipose *Pref. 1* gene was negatively associated with plasma MIF levels (Figure 1H), suggesting that Pref-1 expression in WAT may negatively modulate the release of adipose MIF and influence plasma MIF levels (Figure 1F).

To investigate whether Pref-1 mediates non-inflammatory MIF release, we employed a non-inflammatory mouse model of obesity. Wild-type mice (WT, 20-week-old) were fed a high palmitic acid diet (PD) for eight weeks, which induced obesity (Figure S3) without altering either the gene expression of the pro-inflammatory (M1) macrophage markers (*Tnfa* [TNF-α], *Il1b* [IL-1β] and *Il6* [IL-6]) or the macrophage polarization (M1/M2) ratio in subcutaneous or visceral adipose tissues (Figures S4, 1I, and 1J). In comparison to their counterparts fed a normal chow (NC) diet, these obese mice exhibited IR (Figure 1K) that was accompanied by a downregulation of Pref-1 gene and protein expression in abdominal adipose tissue (Figures S5A, 1L and 1M). The reduced Pref-1 expression was not associated with any change in the expression of adipogenetic genes, such as *Fabp4* and *PPARγ* (Figure 1L) or the number of Pref-1+ cells (Figures 1N and S5B). In the group fed PD, Pref-1 release from isolated WATs was significantly lower than in the group fed NC (Figures 1N and S5C). The reduction of Pref-1 expression and release was not associated with changes in *Mif* gene expression (Figure 1O), however, there was a decrease in adipose MIF protein content (Figures 1P and 1Q) that was associated with an increase in circulating plasma MIF levels (Figure 1R). Pref-1 is cleaved at the cell membrane leading to the release of its active component.^17^ To further define whether Pref-1 regulates adipose MIF release, isolated WATs from NC and PD groups were treated with or without recombinant, bioactive Pref-1 protein (rPref-1, 2.5 μg/mL) for 24 h. The PD group demonstrated increased MIF release compared to the NC group; this was inhibited by rPref-1 (Figure 1S), suggesting a crucial role of Pref-1 in inhibiting MIF release from WAT.

In order to better define the role of circulating MIF in PD-induced IR, we assessed the actions of neutralizing anti-MIF antibody administration. Anti-MIF significantly improved whole-body and adipose insulin sensitivity and attenuated obesity in PD group (Figures 1T and S6). In reciprocal experiments, increasing plasma MIF concentrations in a mouse model, overexpressing MIF in lung (*Mif* lung Tg mice (*Mif* Tg)), demonstrated that high circulating MIF levels (Figure 1U) are associated with IR and hypertriglyceridemia in the absence of alterations in inflammatory gene expression in adipose tissue (Figures 1V, 1W, and S7). These data together support the role of MIF in regulating IR in the absence of adipose inflammation.

### Pref-1 inhibits MIF release from both adipose Pref-1+ cells and adipocytes

**Figure 2:**
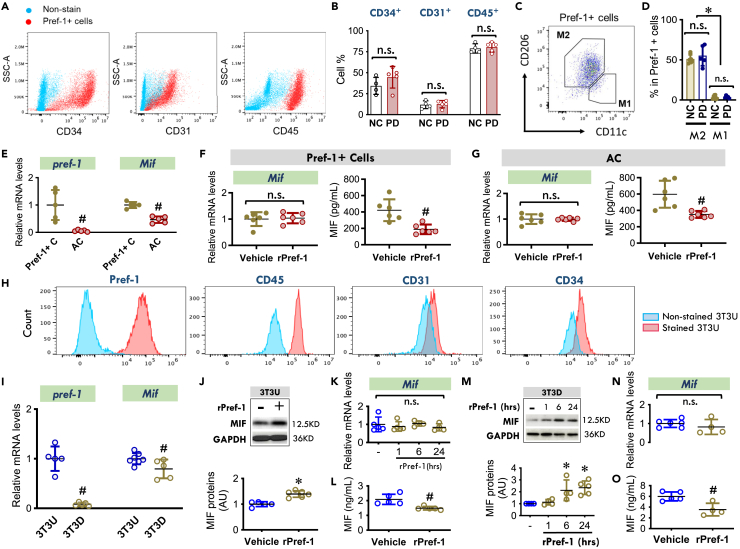
Pref-1 inhibits MIF release from both adipose Pref-1+ cells and adipocytes (A) Cells with CD34^+^, CD31^+^, or CD45^+^ in Pref-1+ cells isolated from WAT in 20-week-WT mice by flow cytometry. (B) The percentages of CD34^+^, CD31^+^, or CD45^+^ cells in Pref-1+ cells following 8-week-NC or PD diet feeding. (C and D) M1 macrophages (CD45^+^F4/80^+^CD11b^+^CD11c^+^) and M2 macrophages (CD45^+^F4/80^+^CD11b^+^CD206^+^) were identified in Pref-1+ cells from NC and PD groups by flow cytometry. (E) Gene expression of *pref-1* and *Mif* in isolated Pref-1+ cells (Pref-1+ C) and adipocytes (AC) quantified by qPCR. (F and G) *Mif* gene expression and its release in isolated Pref-1+ cells and adipocytes following the treatment of mouse recombinant Pref-1 protein (rPref-1, 2.5 μg/ml) for 24 h. (H) 3T3-L1 undifferentiated cells were identified with Pref-1, CD45, CD31, and CD34 antibodies by flow cytometry. (I) The genetic quantifications of *pref-1* and *Mif* in 3T3-L1 undifferentiated (3T3U) and differentiated (3T3D) cells. (J–O) 3T3U and 3T3D were incubated with or without rPref-1 for 24h and MIF protein content (J and M), gene expression (K and N) and release (L and O) were measured by western blot, qPCR and ELISA, respectively. All data are presented as mean ± SD. ∗p ≤ 0.05 increase and ^#^p ≤ 0.05 reduction vs. Vehicle. The n.s. represents no significance.

We isolated the cell populations that express and potentially release Pref-1 in WAT. Most of these cells co-stained with CD34, CD31, and CD45 (Figure 2A), suggesting the characteristics of progenitor cells (*e.g.,* CD34), endothelial cells (*e.g.,* CD31), and immune cells (e.g., CD45). The components of CD34^+^, CD31^+^, and CD45^+^ cells in the total Pref-1+ cells were unchanged following high PD feeding (Figure 2B). We also identified that M2 rather than M1 macrophages express Pref-1 (Figures 2C and 2D). PD feeding did not affect the proportion of the M2 cells present among the Pref-1+ population of cells (Figure 2D).

Although, both Pref-1+ cells and mature adipocytes isolated from WAT express MIF, MIF expression was lower in the mature adipocytes (Figure 2E). Pref-1 is associated with inflammatory factors in obese humans in both subcutaneous and omental adipose tissue.^21^ We tested whether secreted Pref-1 protein may regulate MIF release from Pref-1+ cells and adipocytes. Following treatment with rPref-1 protein (2.5 μg/mL for 24h), both cell types showed reduced MIF secretion in the absence of a change in *Mif* mRNA content (Figures 2F and 2G), implicating Pref-1 in the cross talk between Pref-1+ cells and adipocytes and its action to inhibit MIF release.

Pref-1 was originally cloned from 3T3-L1 precursor fibroblasts, which also express CD45, CD31, and CD34 (Figure 2H) as isolated adipose Pref-1+ cells (Figure 2A). *Mif* mRNA expression was reduced when the precursor fibroblasts (Pref-1+) were differentiated into adipocytes (Figure 2I), which mimicked the pattern of *Mif* expression observed in Pref-1+ cells versus mature adipocytes in healthy mouse WAT (Figure 2E). Following rPref-1 treatment of 3T3-L1 precursor fibroblasts, we observed a significant increase in MIF protein but not *Mif* mRNA content (Figures 2J and 2K). Interestingly, MIF concentration in conditioned media was reduced after Pref-1 treatment (Figure 2L). We also investigated the possible paracrine role of Pref-1 in regulating MIF release from 3T3-L1 adipocytes (AC). Following recombinant Pref-1 treatment, MIF protein content increased in 3T3-L1 differentiated ACadipocytes in a time-dependent manner (Figure 2M), mimicking the observations in precursor cells that an increase in MIF protein is not associated with changes in *Mif* mRNA levels (Figure 2N), but rather with reduced MIF release into the medium (Figure 2O).

Based on these findings in primary cells and cell lines, we suggest that secreted Pref-1 inhibits MIF release from both Pref-1+ cells and mature adipocytes in an autocrine/paracrine manner.

### Pref-1 inhibits MIF secretion by interaction with the cell membrane protein, integrin 1β, and inhibiting p115, a cofactor for MIF release

**Figure 3:**
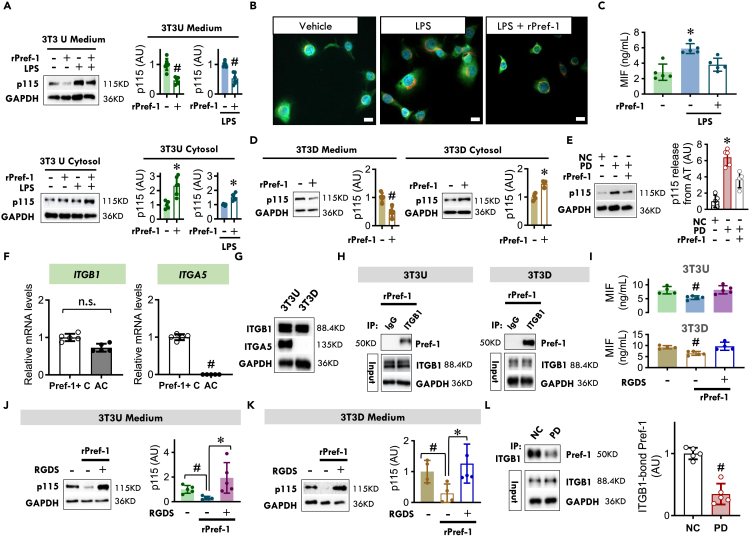
Pref-1 inhibits MIF secretion by interaction with the cell membrane protein, integrin 1β, and inhibiting p115, a cofactor for MIF release (A) 3T3-L1 undifferentiated (3T3U) Pref-1+ cells were treated with or without rPref-1 (2.5 μg/ml) in the absence or presence of LPS (5 μg/ml) for 24 h p115 was then quantified in cytosol and medium by western blot. (B) MIF (green) and p115 (red) were visualized by immunofluorescence staining in 3T3-L1 undifferentiated Pref-1+ cells following vehicle or LPS treatment. The nucleus was stained by DAPI (blue), (Scale bars, 10 μm). (C) MIF release from 3T3-L1 undifferentiated Pref-1+ cells following vehicle or LPS treatment in the presence or absence of rPref-1. (D) p115 in cytosol and medium of 3T3-L1 differentiated adipocytes (3T3D) quantified by western blot following rPref-1 treatment for 24 h. (E) p115 release from WATs isolated from NC and PD groups in the presence of absence of rPref-1 (2.5 μg/ml). (F) The gene expression of ITGB1 and ITGA5 in Pref-1+ cells and adipocytes isolated from WT visceral adipose tissue. (G) ITGB1 and ITGA5 genes in 3T3U and 3T3D. (H) In 3T3U and 3T3D, immunoprecipitation was performed with negative-control antibody (IgG) or anti-ITGB1 following the treatment of rPref-1. Immunoprecipitated proteins were then detected by anti-Pref-1 antibody. (I–K) MIF and p115 release from 3T3U and 3T3D following the treatment of rPref-1, in the presence or absence of integrin inhibitor, RGDS peptide (100 μM). (L) Following normal chow (NC) or high palmitic acid diet (PD) feeding, visceral adipose tissues isolated from WT mice were initially immunoprecipitated with anti-ITGB1 antibody and the immunoprecipitated proteins were further detected by anti-Pref-1 antibody. All data are presented as mean ± SD. ∗p ≤ 0.05 increase and ^#^p ≤ 0.05 reduction vs. Vehicle, Pref-1+ cells, IgG, NC, or all other groups. The n.s. represents no significance.

To investigate the cellular mechanisms by which Pref-1 regulates MIF release, we examined the 3T3-L1 precursor fibroblast model of adipocyte differentiation. MIF lacks a signal sequence and is secreted from cells by a non-conventional pathway for protein export.^22^ The Golgi-associated protein, p115, is an intracellular binding partner of MIF that is co-secreted with MIF.^23^ The deletion of p115 from monocytes/macrophages reduces the release of MIF but no other cytokines following inflammatory stimulation or intracellular bacterial infection.^23^ We observed that rPref-1 treatment of 3T3-L1 undifferentiated Pref-1+ cells (3T3U) for 24 h significantly reduced the release of p115 (Figure 3A) and MIF (Figure 2L) into medium; this occurred in the absence of inflammatory stimulation and was associated with an increase in intracellular p115 content (Figure 3A). Pref-1 also inhibits lipopolysaccharide (LPS) triggered MIF release (Figure 3C) by inhibiting p115 release (Figure 3A). We studied the cellular distribution of MIF in 3T3-L1 Pref-1+ cells by immunofluorescence staining and found that MIF and p115 appeared to co-localize in the cytoplasm (Figure 3B). Upon LPS stimulation, both MIF and p115 staining was more diffuse in the cytoplasm and staining intensity was reduced following the addition of rPref-1 (Figure 3B), suggesting a role for Pref-1 in inhibiting MIF release by interfering with the cytoplasmic interaction of MIF and p115. In differentiated 3T3-L1 adipocytes (3T3D), Pref-1 also reduced basal p115 release, which was associated with an increase in cytosolic p115 content (Figure 3D). The reduction in p115 release is associated with reduced MIF release (Figure 2O), which is consistent with prior observations describing the role of p115 in MIF export.^23^ In parallel with changes with MIF release (Figure 1S), the PD fed group demonstrated increased adipose p115 release when compared to the NC group, and this effect was reduced following rPref-1 treatment (Figure 3E). Taken together, these data suggest that Pref-1 inhibits MIF release by the intermediation of p115 in both Pref-1+ cells and adipocytes in WAT.

Pref-1 signaling is associated with the activation of the classic fibronectin receptor, α5β1 integrin.^24^ β1 integrin (ITGB1) is highly expressed in both Pref-1+ cells and adipocytes but α5 integrin (ITGA5) is only expressed in Pref-1+ cells (Figures 3F and 3G). Given the evidence that Pref-1 regulates MIF release in both cell types (Figure 2), we hypothesized that ITGB1 activation may be an essential component in Pref-1 regulation of MIF release. Indeed, Pref-1 binds to ITGB1 in both 3T3 undifferentiated Pref-1+ cells and 3T3 differentiated adipocytes (Figures 3H and S8). The inhibition of integrin receptor function significantly reversed Pref-1 inhibition of MIF and p115 release from both cell types (Figures 3I–3K). In mice, PD feeding significantly reduced adipose Pref-1 expression and release (Figures 1L–1N) when compared to the NC group, and this effect was associated with a reduction in the binding of Pref-1 with ITGB1 (Figure 3L). The data together suggest that Pref-1 regulates MIF release from WAT through binding with ITGB1 in both Pref-1+ cells and adipocytes.

### PAR2 activation and expression modulate Pref-1 in an AMPK-dependent manner in Pref-1+ cells

**Figure 4:**
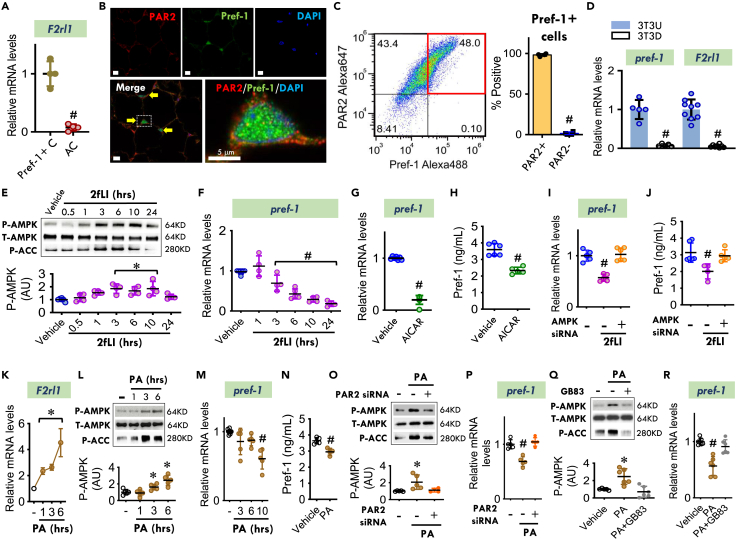
PAR2 activation and expression modulate Pref-1 in an AMPK-dependent manner in Pref-1+ cells (A) The mRNA expression of *F2rl1* (PAR2) and *Mif* in isolated Pref-1+ cells (Pref-1+ C) and adipocytes (AC) from 25-week-old WT mouse visceral adipose tissue. (B) Colocalization of PAR2 (red) and Pref-1 (green) in WT mouse visceral adipose tissue. (Arrows indicate colocalization. Yellow scale bars, 20 μm, white scale bar: 5 μm). (C) Scatter-dot plot and quantification of flow cytometry analysis of PAR2 and Pref-1 in mouse stromal vascular fraction (SVF) cells. (D) The *pref-1* and *Par2* transcript levels were quantified in 3T3-L1 undifferentiated (3T3U) and fully differentiated (3T3D) cells. (E and F) AMPK activation (E) and *pref-1* mRNA expression (F) in 3T3-L1 undifferentiated cells cultured with 2fLI (30 nM) for 0.5–24 h. (G and H) Pref-1 mRNA expression (10 h) (G) and release in the cell media (24 h) (H) in 3T3-L1 undifferentiated cells incubated with AICAR (0.25 mM) for 10–24 h. (I and J) Pref-1 expression (I) and release (J) in 3T3-L1 undifferentiated cells (Pref-1+ cells) cultured with or without 2fLI (30 nM) for 24 h following knockdown of AMPKα1 and α2 isoforms by siRNA. (K–N) *F2rl1* (PAR2) mRNA expression (K), AMPK phosphorylation (L), *pref-1* mRNA expression (M), and Pref-1 release (24 h) (N) in 3T3-L1 undifferentiated cells (Pref-1+ cells) incubated with high palmitic acid (PA, 200 μM) for 1–24 h. (O and P) AMPK activity (6 h) (O) and *pref-1* mRNA expression (10 h) (P) in 3T3-L1 undifferentiated cells incubated with or without high PA treatment following knockdown of PAR2 by siRNA. (Q and R) AMPK activity (6 h) (Q) and *pref-1* mRNA expression (10 h) (R) in 3T3-L1 undifferentiated cells following high PA treatment with or without the PAR2 inhibitor, GB83 (5 μM). All data are presented as mean ± SD. 2-tailed Student’s *t* test in (A), (C), (D), (G), (H), and (N), and one-way ANOVA plus Tukey in the rest of data were used for statistical analysis. ∗p ≤ 0.05 increase vs. Vehicle in (E) and (L); vs. other groups in (O) and (Q); ^#^p ≤ 0.05 reduction vs. Pref-1+ C in (A), PAR2+ in (C) and 3T3U in (D); vs. Vehicle in (F), (G), (M), and (N), vs. other groups in (I), (J), (P), and (R).

Protease-activated receptor 2 (PAR2) is a unique G-protein coupled receptor that is encoded by the *F2rl1* gene. *F2rl1* is highly expressed in isolated Pref-1+ cells rather than adipocytes (Figure 4A). In Pref-1+ cells, PAR2 protein was expressed peripherally and along the plasma membrane, while Pref-1 showed a diffuse cytosolic staining pattern (Figure 4B). Quantitative flow cytometry of freshly isolated stromal vascular fraction (SVF) cells showed that almost all Pref-1+ cells were also PAR2 positive (Figures 4C and 4D) confirming that Pref-1+ cells express PAR2.

PAR2 activation leads to downstream phosphorylation of AMPK at its Thr^172^ activation site.^25^ The pharmacologic activation of PAR2 with the PAR2 activating peptide, 2-furoyl-LIGRLO-NH2 (2 fLI, 30 nM) increased AMPK phosphorylation in a time-dependent manner in Pref-1+ cells (Figure 4E). The *pref-1* mRNA expression was downregulated over the same time frame (Figure 4F). Activating AMPK with the activator 5-Aminoimidazole-4-carboxamide ribonucleotide (AICAR) (Figure S9A) also downregulated *pref-1* mRNA expression in Pref-1+ cells (Figure 4G). AMPK-mediated downregulation of *pref-1* in turn reduced Pref-1 release from Pref-1+ cells (Figure 4H). Knocking down AMPK (Figure S9C) in Pref-1+ cells prevented the PAR2-induced reduction in *pref-1* expression (Figure 4I) and Pref-1 release (Figure 4J). These findings establish a requisite role for AMPK in PAR2-mediated *pref-1* downregulation in Pref-1+ cells. Pref-1 downregulates *PPAR*γ expression which impedes differentiation of adipocyte precursor cells into mature adipocytes.^17^ However, when we examined *PPAR*γ expression, we found no change in *PPAR*γ levels in 3T3-L1 undifferentiated cells despite activation of AMPK and reduced *pref-1* expression (Figures S9B and S9D). Thus, these results indicate that PAR2-induced regulation of AMPK and *pref-1* expression occurs independently of *PPAR*γ expression.

PD upregulates PAR2 expression in adipose tissue (Figure S10). We investigated whether high palmitic acid (PA) concentrations modulate the PAR2/AMPK/Pref-1 pathway in Pref-1+ cells. Undifferentiated 3T3-L1 cells incubated with a high concentration of PA (200 μM) upregulated *F2rl1* (PAR2) expression (Figure 4K) in conjunction with activation of downstream extracellular signal-regulated kinase (ERK) phosphorylation (Figure S11A). PA also increased AMPK phosphorylation (Figure 4L), which was accompanied by a reduction in *pref-1* gene expression and Pref-1 release into the culture media (Figures 4M and 4N). PA addition, however, did not alter *PPAR*γ gene expression in these Pref-1+ cells (Figure S11B). To examine whether PAR2 activation had an obligatory role in palmitic acid-induced AMPK activation and downstream inhibition of *pref-1* expression, undifferentiated 3T3-L1 cells were treated with high PA in the presence of the PAR2-specific inhibitor GB83 (5 μM) or following PAR2 knockdown by siRNA. Both pharmacologic and siRNA inhibition of PAR2 abolished AMPK activation and blocked the PA treatment induced reduction in *pref-1* gene expression (Figures 4O–4R).

### PAR2 controls MIF release from Pref-1+ cells and adipocytes through Pref-1

**Figure 5:**
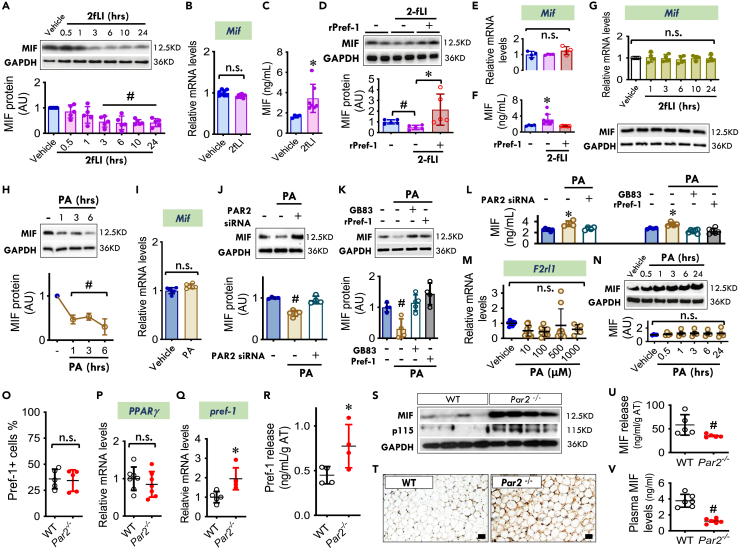
PAR2 controls MIF release from Pref-1+ cells and adipocytes through Pref-1 (A–C) MIF protein (A), mRNA expression (24 h) (B) and release in the medium (24 h) (C) following PAR2 activator, 2fLI (30 nM) treatment for 0.5–24 h in 3T3-L1 undifferentiated cells. (D–F) MIF protein (D), mRNA expression (E), and release levels (F) following 2-fLI treatment (30 nM) with or without rPref-1 (2.5 μg/mL) for 24 h. (G) MIF mRNA and protein expression following 2fLI (30 nM) treatment for 1–24 h in 3T3-L1 differentiated cells. (H and I) MIF protein (H) and gene expression (I) in 3T3-L1 undifferentiated cells (Pref-1+ cells) following high palmitic acid (PA, 200 μM) treatment for 24h. (J–L) Following knocking down PAR2 with siRNA or inhibition of PAR2 with its antagonist, GB83 (5 μM), cellular MIF levels were quantified in the presence or absence of high PA with or without rPref-1 (J and K). MIF release in the medium was measured by ELISA in (L). (M and N) *F2rl1* (PAR2) gene (M) and MIF protein (N) levels in differentiated 3T3-L1 adipocytes following variable concentrations of PA for 24 h. (O–Q) In 25-week WT and *Par2*^*−/−*^ mice, the percentages of adipose Pref-1+ cells were quantified by flow cytometry (O). The gene expression of *PPARγ* and *pref-1* was evaluated by qPCR (P and Q). (R–V) Visceral adipose tissues isolated from WT and *Par2*^*−/−*^ mice were cultured in PBS for 24 h at 37°C. Pref-1 and MIF proteins were assessed by ELISA in the medium (R and U) while cellular MIF and p115 levels were measured by western blot (S) and immunohistochemistry staining (Scale bars, 20 μm) (T). Plasma MIF levels were quantified by ELISA (V). All data are presented as mean ± SD. 2-tailed Student’s *t* test in (B), (C), (I), (O-R), (U), and (V), and one-way ANOVA plus Tukey in the rest of data for statistical analysis. ∗p ≤ 0.05 increase vs. Vehicle in (C); vs. other groups in (F) and (L); vs. 2FLI+Vehicle in (D); vs. WT in (Q) and (R). ^#^p ≤ 0.05 reduction vs. Vehicle in (A), (D) and (H); vs. other groups in (J) and (K); vs. WT in (U) and (V). The n.s. represents no significance.

We further investigated whether PAR2 regulates MIF release. We treated 3T3-L1 undifferentiated Pref-1+ cells with 2 fLI (30 nM) and observed decreased MIF protein content (Figure 5A) but no change in *Mif* mRNA levels (Figure 5B). In parallel, we found that MIF content in the media was significantly increased following the addition of 2fLI (Figure 5C). These findings suggest that PAR2 may augment cellular MIF release in Pref-1+ cells without altering *Mif* gene expression, thereby reducing intracellular MIF content. Pref-1 inhibits MIF release from Pref-1+ cells and thus, Pref-1 may mediate the functional interaction between the PAR2 and MIF pathway. To test this hypothesis, we activated the PAR2 pathway pharmacologically with 2-fLI in the presence or absence of Pref-1 in Pref-1+ cells. PAR2 activation enhanced MIF release and reduced cellular MIF content, and these effects were reversed by the addition of rPref-1 (Figures 5D and 5F). *Mif* gene expression remained unchanged (Figure 5E). This observation additionally appeared only in Pref-1+ cells. In contrast, MIF expression and content were not affected by 2fLI in 3T3-L1 differentiated Pref-1 negative adipocytes (Figure 5G), which have negligible *Par2* expression (Figure 4D).

With increased PAR2 expression following high PA treatment (Figure 4K), MIF content in 3T3-L1 undifferentiated Pref-1+ cells was decreased in a time-dependent manner (Figure 5H) and this reduction occurred without changes in *Mif* gene expression (Figure 5I). To examine whether PA-downregulated MIF content requires PAR2 expression and activation, PAR2 knockdown and inhibition were performed. Both siRNA and pharmacologic inhibition of PAR2 reversed palmitic acid-induced depletion of cellular MIF and this inhibition was blocked by the addition of rPref-1 (Figures 5J and 5K). In parallel, high PA augmented MIF release, but this effect of PA could be reversed by blockade of PAR2 (Figure 5L). In contrast, in differentiated 3T3-L1, high PA did not trigger *F2rl1* (PAR2) gene expression or alter cellular MIF content (Figures 5M and 5N).

We also found that in the absence of PAR2 expression, there was no evident alteration in either adipose Pref-1+ cell populations and characteristics or PPARγ expression (Figures 5O, 5P, and S12), however, there was an increase in both *pref-1* mRNA expression (Figure 5Q) and Pref-1 release (Figure 5R). PAR2 deficiency upregulated adipose content of MIF and p115 (Figures 5S and 5T), which was associated with a decrease in both MIF release from WAT (Figure 5U) and circulating MIF levels (Figure 5V). These *in vivo* data further suggest that the PAR2/Pref-1 signaling pathway may play a key role in regulating MIF accumulation and release from WAT even in the absence of inflammation (Figure S13).

PAR2 deficiency in WAT or addition of Pref-1 decreases non-inflammatory adipose MIF release and improves insulin sensitivity.

**Figure 6:**
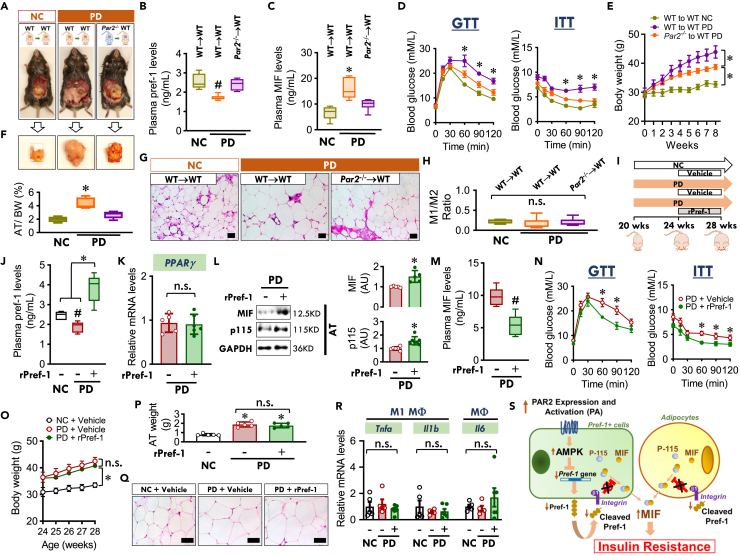
PAR2 deficiency in WAT or addition of Pref-1 decreases non-inflammatory adipose MIF release and improves insulin sensitivity (A) WT mice at 18 weeks were anesthetized and transplanted with WT or *Par2*^*−/−*^ visceral epididymal adipose tissue. Following 2-week recovery, these mice at 20 weeks will be fed with normal chow (NC) or high palmitic acid diet (PD) for 8 weeks. (B–H) Plasma Pref-1 (B) and MIF (C) levels in the mice transplanted with WT or *Par2*^*−/−*^ adipose tissue following NC or PD diet feeding. Insulin resistance was assessed by GTT and ITT (D). Body weight gain, adipose weight (AT)/body weight (BW) ratio, adipocyte size, and M1/M2 ratio in adipose tissue were measured in these mice as well (E to H) (Scale bars, 20 μm in G). (I) WT mice at 20 weeks were fed with NC or PD for 8 weeks. During the last 4 weeks, the mice were infused with vehicle or recombinant Pref-1 protein (rPref-1, 24 μg/day/kg) by osmotic pump. (J–R) Plasma Pref-1 levels, PPARγ gene expression, adipose MIF and p115 contents, and plasma MIF levels were subsequently evaluated from (J) to (M). Insulin resistance was assessed by GTT and ITT in (N). Body weight gain, adipose tissue weight, adipocyte size, and expression of inflammatory factors were quantified from (O) to (R) (Scale bars, 20 μm in Q). (S) Schematic diagram for the mechanism of PAR2/AMPK/Pref-1/MIF release signaling pathway. Pref-1 is expressed and released in Pref-1+ cells with characteristics of M2 macrophages, endothelial cells or progenitors. Pref-1 inhibits MIF release from both Pref-1+ cells and adipocytes by binding with cell membrane integrin β1 and inhibiting the mobilization of p115, a cofactor for MIF release. High palmitic acid (PA) induces PAR2 expression in Pref-1+ cells, leading to the downregulation of Pref-1 expression and its release in an AMPK-dependent manner. When Pref-1 secretion is reduced, Pref-1+ cells and adipocytes increase MIF release and its plasma content, resulting in insulin resistance. All data are presented as mean ± SD. n = 6 each animal group. ∗p ≤ 0.05 increase vs. other groups in (C, D, F and J); vs. WT to WT NC or *Par2*^*−/−*^ to WT PD in (E); vs. NC in (O) and (P). ^#^p ≤ 0.05 reduction vs. other groups in (B); vs. NC in (J); vs. PD + Vehicle in (M) and (N). The n.s. represents no significance.

We next transplanted visceral (epididymal) adipose tissue from WT to WT, *Par2*^*−/−*^ to WT or WT to *Par2*^*−/−*^ mice, respectively (Figures 6A, S14, and S15).^26^ Following a PD feeding, plasma Pref-1 levels were significantly reduced in the group of WT to WT while *Par2*^*−/−*^ adipose tissue transplantation into WT mice reversed the effect of PD (Figure 6B). In parallel, PD increased plasma MIF levels, IR, body weight and adipose tissue mass, and these effects were all inhibited by *Par2*^*−/−*^ adipose tissue transplantation into WT mice (Figures 6C–6G). Additionally, we found no evidence of adipose inflammation after PD feeding, indicating that this was a non-inflammatory mechanism (Figure 6H).

In an additional separate experiment, 20-week-old WT mice were initially fed with either NC or a high PD for 4 weeks. The mice were implanted subsequently with an osmotic pump filled with vehicle or rPref-1 for 4 weeks of infusion with continued PD feeding (Figures 6I and S16). We observed that administration of Pref-1 significantly blocked the reduction of plasma Pref-1 levels induced by PD feeding (Figure 6J). There were no associated changes in *PPARγ* expression (Figure 6K) to suggest that infusion of Pref-1 might affect adipogenesis. However, Pref-1 administration augmented the adipose content of MIF and p115 (Figure 6L) and circulating plasma MIF levels were reduced (Figure 6M). These results suggested that exogenous Pref-1 infusion inhibits MIF release from adipose tissue. Pref-1 infusion attenuated IR during PD (Figure 6N), despite no evident change in body weight gain, adipose tissue weight, and adipocyte size compared to the PD group with vehicle infusion (Figures 6O–6Q). As in the study of *Par2*^*−/−*^ adipose tissue transplantation, Pref-1 infusion did not affect inflammatory gene expression (Figure 6R). These findings indicate that Pref-1 treatment reverses non-inflammatory IR by downregulating adipose MIF release.

In summary, these data support a novel, inflammation-independent PAR2/Pref-1/MIF pathway that acts between Pref-1+ cells and mature adipocytes to regulate WAT MIF release in obesity related IR (Figure 6S).

## Discussion

Accumulating evidence suggests that non-inflammatory mechanisms may initiate IR.^5^^,^^27^ We describe a Pref-1 mediated pathway involving the crosstalk between cells expressing Pref-1 (Pref-1+) and adipocytes that regulates MIF secretion from adipose tissue in response to high PA exposure. The molecular pathway underlying this crosstalk is initiated by PAR2, whose expression is induced by high fat diet; PAR2 downregulates Pref-1 expression and release from Pref-1+ cells in an AMPK-dependent manner. Normally, Pref-1, derived from Pref-1+ cells, inhibits MIF release from both Pref-1+ cells and adipocytes by binding to β1 integrin and inhibiting the action of p115, a necessary cofactor for MIF secretion. However, in the context of non-inflammatory obesity, the ability of Pref-1 to inhibit MIF release from Pref-1+ cells and adipocytes is impaired; consequently, circulating plasma MIF levels are increased. This finding is significant given MIF’s maladaptive action to cause metabolic dysfunction and IR in obesity, as evidenced in experimental results in mice as well as human subjects with functionally variant *MIF* alleles.^14^^,^^28^^,^^29^

WAT is known to be an important site of MIF production, and circulating MIF levels correlate positively with increased body weight.^10^ Early studies attributed the upregulation of adipose MIF secretion to macrophage infiltration.^13^^,^^14^ One prior study reported that high caloric diet feeding induces an increase in adipose macrophages and *Mif* gene expression, leading to an elevation in plasma MIF levels.^14^ In metabolic dysfunction associated with treatment with the antipsychotic olanzapine, high plasma MIF levels are accompanied by an increase in both MIF gene and protein expression in WAT.^29^ Increased MIF expression also was associated with increased expression of IL-6 and IL-1β,^29^ further supporting an inflammatory etiology for adipose MIF secretion with olanzapine treatment. In contrast, the current study provides the first evidence for a non-inflammatory pathway of MIF secretion in WAT. Animals fed a high fat diet for 8 weeks showed increased MIF secretion by WAT in the absence of increased *Mif* mRNA expression or a significant, associated inflammatory response. Thus, our findings support the existence of a mechanism of MIF secretion that is metabolically controlled and independent of adipose macrophage infiltration or inflammatory activation.

The current results also demonstrate that non-inflammatory MIF release from WAT is mediated by cellular cross talk driven by Pref-1. Pref-1 was expressed and released by Pref-1+ cells, which included M2 macrophage (an anti-inflammatory phenotype), endothelial cell, and progenitor populations. Pref-1 inhibited MIF release from both Pref-1+ cells and adipocytes. Accordingly, the activation and secretion of Pref-1 may reduce non-inflammatory MIF secretion from WAT and improve metabolic dysfunction. Indeed, *pref-1* overexpression improved glucose homeostasis and insulin sensitivity.^20^ It should be noted that Pref-1 was originally identified to regulate adipogenesis.^17^ Mice with high levels of Pref-1 in adipose tissue have reduced fat mass and hypertriglyceridemia due to impaired storage in adipose tissue.^18^ In contrast, mice lacking Pref-1 show augmented fat deposition and obesity.^19^ However, our current findings indicate that while Pref-1 expression and release by WAT were downregulated following high fat feeding, this occurred in the absence of changes in adipose maturation. Thus, the effect of Pref-1 described herein reflects an early phase of metabolic dysfunction; and it is possible that actions on adipogenesis may be detected in a more chronic obesity model.

We found that Pref-1 significantly inhibited basal and/or LPS-induced MIF release from Pref-1+ cells and adipocytes through p115. The Golgi-associated protein, p115 is a binding partner of MIF that facilitates MIF transport from the perinuclear ring to the plasma membrane and subsequent export from monocytes/macrophages.^23^ p115 is necessary for the release of MIF but not for other cytokines.^23^ Pref-1 signaling is associated with the activation of the classic fibronectin receptors, α5 and β1 integrins,^24^ but only β1 integrin (ITGB1) is highly expressed in both Pref-1+ cells and adipocytes. Our current findings indicated that Pref-1 modulates p115 and MIF release by binding to ITGB1 in both Pref-1+ cells and adipocytes. Thus, a Pref-1/ITGB1/p115 pathway appears to be a key mechanism that regulates non-inflammatory MIF release from WAT, leading to high fat-induced IR. Indeed, infusion of rPref-1 in mice significantly upregulated the cytosolic content of MIF and p115 in WAT, and that this effect was associated with a reduction in plasma MIF levels and decreased IR. The MIF release and insulin sensitivity modulated by Pref-1 did not appear to be related to adipose inflammation, further supporting our hypothesis that MIF is a key regulator of non-inflammatory mechanisms of IR.

Our findings also demonstrate that the expression and activation of PAR2 negatively regulates Pref-1 expression and release from Pref-1+ cells in WAT. PAR2 is a seven transmembrane receptor expressed by endothelial cells and macrophages.^30^^,^^31^ We also showed that PAR2 is highly expressed in Pref-1+ cells rather than adipocytes. PAR2 is known to activate AMPK through Ca^2+^/CaMKKβ signaling,^25^ and we found that AMPK is required for the PAR2-mediated downregulation of both *pref-1* expression and Pref-1 release in Pref-1+ cells in adipose tissue. Thus, the absence of PAR2 expression in WAT significantly increased Pref-1 expression and release. PAR2 expression was upregulated by PA. Following short-term treatment with a high PA diet, PAR2 expression and activation in Pref-1+ cells occurred without concomitant alterations in adipose cytokine gene expression, and pharmacologic activation of PAR2 in turn stimulated MIF release rather than *Mif* gene expression. Downregulation of Pref-1 following PAR2 activation prevented its inhibitory action on MIF release in Pref-1+ cells and adipocytes, leading to increased plasma MIF levels. We therefore conclude that PAR2/Pref-1 mediates a novel pathway for autocrine/paracrine signaling between Pref-1+ cells and adipocytes that influences circulating MIF levels in the absence of inflammation.

Recent experimental work is also consistent with the hypothesis that IR occurs prior to systemic or adipose inflammation.^5^ Clinical research indicates that adipose inflammation is not causally linked to IR^27^ and targeting TNFα fails to provide beneficial effect on systemic insulin sensitivity.^32^ Immunocompromised animal models are also not protected from IR induced by a short-term high fat diet.^33^ Thus, elucidating the proximate role of non-inflammatory mechanisms in the initiation of IR is potentially important. We found that alterations in Pref-1 in the regulation of adipose MIF secretion may have an early role in the development of high fat-induced IR prior to the inflammatory activation of adipose tissue macrophages. Furthermore, PAR2 appears to be critical in the inhibition of Pref-1 secretion. These data together suggest that Pref-1 and its upstream regulator, PAR2, could be a tractable therapeutical target for early, non-inflammatory IR. Whether PAR2 has additional distinct actions on lipid metabolism independent of its effects on Pref-1 and MIF against obesity will also be of interest for further studies.

In conclusion, we have identified a novel autocrine/paracrine mechanism mediated by secreted Pref-1 that contributes to high fat-induced metabolic dysfunction and is independent of adipose macrophage infiltration and inflammation. PAR2 is highly expressed in various adipose tissues, including subcutaneous and visceral adipose tissues.^34^ PAR2 expression and activation downregulate Pref-1 expression and release from Pref-1+ cells in WAT. Strategies to block fatty acid-induced PAR2 expression and/or to augment Pref-1 expression, activation or secretion may reduce adipose tissue MIF secretion and improve metabolic dysfunction. Further work is warranted to investigate the therapeutic applicability of these strategies in the clinical settings of obesity and type 2 diabetes.

### Limitations of the study

The focus of this study was to identify the mechanisms underlying IR in the absence of inflammation. We found that pref-1 regulates non-inflammatory MIF release from adipose tissue, which was previously recognized as a risk factor for IR. However, although PAR2 and Pref-1 co-localize in Pref-1+ cells, our study did not demonstrate the role of PAR2 in mediating Pref-1 expression and release in Pref-1+ cells by specifically knocking down PAR2 in Pref-1+ cells in animal models.

## STAR★Methods

### Key resources table

**Table:** 

REAGENT or RESOURCE	SOURCE	IDENTIFIER
**Antibodies**		

Rabbit-anti-GAPDH	Cell Signaling Technology	2118S
Rabbit-anti-P-Akt (Ser473)	Cell Signaling Technology	9271S
Rabbit-anti-Akt	Cell Signaling Technology	9272S
Rabbit-anti-P-AMPK Alpha (Thr172)	Cell Signaling Technology	2535S
Rabbit-anti-AMPK Alpha	Cell Signaling Technology	2532S
Rabbit-anti-PAR-2	abcam	ab180953
Rabbit-anti-MIF	abcam	ab187064
Rabbit-anti-P115	Proteintech	13509-1-AP
Rat-anti-ITGB1	Invitrogen	Cat# 14-0292-82; RRID: AB_914295
Rabbit-anti-ITGB1	Invitrogen	Cat# PA5-78028; RRID: AB_2735967
Rabbit-anti-ITGA5	Invitrogen	Cat# PA5-82027; RRID: AB_2789188
Mouse-anti-Pref-1	Invitrogen	Cat# MA5-15915; RRID: AB_11155588
Goat-anti-Pref-1	R&D SYSTEMS	AF8277
Rabbit-anti-Pref-1	R&D SYSTEMS	MAB8634
Mouse-anti-MIF	R&D SYSTEMS	MAB2892
Rabbit-anti-MIF	Torrey Pines Biolabs	TP234
Anti-rabbit IgG, HRP-linked Antibody	Cell Signaling Technology	7074S
Anti-mouse IgG, HRP-linked Antibody	Cell Signaling Technology	7076S
Donkey anti-Goat IgG (H+L) Cross-Adsorbed Secondary Antibody, Alexa Fluor 594	Invitrogen	Cat# A-11058; RRID: AB_2534105
Donkey anti-Goat IgG (H+L) Cross-Adsorbed Secondary Antibody, Alexa Fluor 488	Invitrogen	Cat# A-11055; RRID: AB_2534102
Donkey anti-Rabbit IgG (H+L) Highly Cross-Adsorbed Secondary Antibody, Alexa Fluor 594	Invitrogen	Cat# A-21207; RRID: AB_141637
Goat anti-Rabbit IgG (H+L) Cross-Adsorbed Secondary Antibody, Alexa Fluor 647	Invitrogen	Cat# A-21244; RRID: AB_2535812
Anti-Mouse Alexa Fluor® 647	Jackson ImmunoResearch	Cat# 715-605-150; RRID: AB_2340862
Alexa Fluor® 488 AffiniPure Donkey Anti-Rabbit IgG (H+L)	Jackson ImmunoResearch	Cat# 711-545-152; RRID: AB_2313584
CD45.2-PerCP	eBioscience	Cat# 45-0454-80; RRID: AB_953592
F4/80-PE	eBioscience	Cat# 12-4801-80; RRID: AB_465922
CD11b-APC eFluor 780	eBioscience	Cat# 47-0112-80; RRID: AB_1603195
CD11c-PE-Cy7	eBioscience	Cat# 25-0114-81; RRID: AB_469589
CD31-FITC	Invitrogen	Cat# RM5201: RRID: AB_10373983
CD34-PE	Invitrogen	Cat# MA5-17831; RRID: AB_2539215
CD206-Alexa Fluor™ 488	Invitrogen	Cat# 53-2061-80; RRID: AB_2784748
CD301–Alexa Fluor 647	AbD Serotec	Cat# MCA2392A647T; RRID: AB_872014

**Chemicals, peptides, and recombinant proteins**		

cOmplete^TM^, EDTA-free Protease Inhibitor Cocktail	ROCHE	11873580001
DMEM	Gibco	11965092
Newborn Calf Serum	Gibco	16010159
Fetal Bovine Serum	Gibco	26140079
3-Isobutyl-1-methylxanthine	Sigma-Aldrich	I7018
Insulin	Sigma-Aldrich	I5500
Dexamethasone	Sigma-Aldrich	D4902
Collagenases, Type I	Worthington Biochemical Corporation	LS004196
Bio-Rad Protein Assay Dye Reagent Conc	Bio-Rad	5000006
1,4-Dithiothreitol	ROCHE	10708984001
4x Laemmli Sample Buffer	Bio-Rad	1610747
30% Acrylamide/Bis Solution 29:1	Bio-Rad	1610156
Resolving Gel Buffer	Bio-Rad	1610798
Ammonium persulfate	Sigma-Aldrich	A3678
UltraPure™ Sodium Dodecyl Sulfate	Invitrogen	15525017
TEMED	Invitrogen	15524010
12% Bis-Tris Protein Gels	Invitrogen	NP0349BOX
BLUeye Prestained Protein ladder	Froggabio Inc.	PM007-0500
Immun-Blot PVDF Membrane	Bio-Rad	1620177
Immobilon-PSQ PVDF Membrane	Millipore	ISEQ00010
Clarity Western ECL Subs	Bio-Rad	1705061
Amersham Hyperfilm MP	GE Healthcare	28-9068-45
UltraPure™ Tris Buffer	Invitrogen	15504020
Glycine	Sigma-Aldrich	G8898
TWEEN® 20	Sigma-Aldrich	P7949
SsoAdvanced™ Universal SYBR® Green Supermix	Bio-Rad	1725274
Power SYBR™ Green PCR Master Mix	Applied Biosystem	4367659
iScript cDNA Synthesis Kit	Bio-Rad	1708891
RevertAid H Minus First Strand cDNA Synthesis Kit	Thermo Scientific	K1632
Bovine Serum Albumin	Ge Healthcare	SH3057402
eBioscience™ Intracellular Fixation & Permeabilization Buffer Set	Invitrogen	88-8824-00
Lipofectamine™ RNAiMAX Transfection Reagent	Invitrogen	13778075
TRIzol™ Reagent	Invitrogen	15596018
Chloroform	Sigma-Aldrich	C2432
Isopropanol	Fisher BioReagents	BP26184
Methanol	ACP Chemicals	M-3640
D- (+)-Glucose	Sigma-Aldrich	G7528
HEPES	Sigma-Aldrich	H4034
Ethylenediaminetetraacetic acid (EDTA)	Sigma-Aldrich	EDS
Sodium bicarbonate (NaHCO_3_)	Sigma-Aldrich	S5761
Ethylene glycol-bis(2-aminoethylether)-N,N,N′,N′-tetraacetic acid (EGTA)	Sigma-Aldrich	E3889
Ammonium chloride (NH_4_Cl)	Sigma-Aldrich	A9434
Magnesium sulfate heptahydrate (MgSO_4_)	Sigma-Aldrich	230391
Sodium hydroxide (NaOH)	Sigma-Aldrich	795429
Glycerol	Sigma-Aldrich	G5516
Sodium fluoride (NaF)	Sigma-Aldrich	S7920
β-Glycerol phosphate disodium salt pentahydrate	Sigma-Aldrich	50020
Sodium orthovanadate (Na_3_VO_4_)	Sigma-Aldrich	S6508
Triton™ X-100	Sigma-Aldrich	T8787
Paraformaldehyde	Sigma-Aldrich	158127
Calcium chloride dihydrate (CaCl_2_)	Sigma-Aldrich	223506
Magnesium chloride hexahydrate (MgCl_2_)	Sigma-Aldrich	M9272
Potassium chloride (KCl)	Sigma-Aldrich	P3911
Sodium Chloride (NaCl)	Thermo Scientific	S271-10
Sodium Phosphate Monobasic Anhydrous (NaH_2_PO_4_)	Thermo Scientific	S397-500
Sodium Phosphate Dibasic Anhydrous (Na_2_HPO_4_)	Thermo Scientific	S374-500
GB 83	Axon Medchem	1622
AICAR	Sigma-Aldrich	A9978
2-Furoyl-Leu-Ile-Gly-Arg-Leu-Orn-NH2	Peptides International	PAR-3663-PI
LPS	Invitrogen	00-4976-93
rPref-1	R&D	8545-PR-050
RGDS	R&D	3498/10
Palmitic acid	Sigma-Aldrich	P0500

**Critical commercial assays**		

Mouse Pref-1/DLK-1/FA1 ELISA	RayBiotech	ELM-PREF1-1
Mouse Pref-1/DLK-1/FA1 ELISA Kit	Invitrogen	EM66RB
Mouse MIF DuoSet ELISA	R&D SYSTEMS	DY1978
Human MIF DuoSet ELISA	R&D SYSTEMS	DY289
DuoSet ELISA Ancillary Reagent Kit 2	R&D SYSTEMS	DY008
TG Colorimetric Assay Kit	Elabscience	E-BC-K261-M
Immunoprecipitation Kit	Invitrogen	10007D
L-Type Triglyceride M Enzyme Color A	FUJIFILM Wako Diagnostics	994-02891
L-Type Triglyceride M Enzyme Color B	FUJIFILM Wako Diagnostics	990-02991
Multi-Calibrator Lipid	FUJIFILM Wako Diagnostics	464-01601
HR Series NEFA-HR(2) Color Reagent A	FUJIFILM Wako Diagnostics	999-34691
HR Series NEFA-HR(2) Solvent A	FUJIFILM Wako Diagnostics	995-34791
HR Series NEFA-HR(2) Color Reagent B	FUJIFILM Wako Diagnostics	991-34891
HR Series NEFA-HR(2) Solvent B	FUJIFILM Wako Diagnostics	993-35191
NEFA Standard Solution	FUJIFILM Wako Diagnostics	276-76491

**Experimental models: Cell lines**		

3T3-L1 cell	Store in our lab	N/A

**Experimental models: Organisms/strains**		

C57BL/6J	The Jackson Laboratory	000664
*Par2*^*-/-*^: B6.Cg-*F2rl1*^*tm1Mslb*^/J	The Jackson Laboratory	004993
*Mif Lung Tg*	Dr. Richard Bucala’s lab at Yale University	N/A

**Oligonucleotides**		

Silencer™ Negative Control No. 1 siRNA	Invitrogen	AM4611
*Par2* siRNA	Invitrogen	s65793
AMPK alpha 1/2 siRNA (m)	Santa Cruz Biotechnology	sc-45313
See Tables S1 and S2 for primer sequences	This paper	N/A

**Software and algorithms**		

ImageJ software	NIH	N/A
Prism V8	GraphPad Software	N/A
FlowJo V10	BD	N/A
BioRender	Crunchbase	N/A

**Other**		

Osmotic Pumps Model 1004	ALZET	0009922
Mouse Jugular Catheter Adjustable Length	ALZET	0007701
AIN-93M Purified Diet	ENVIGO	TD.94048
High Palm Oil Diet (93M, G)	ENVIGO	TD.170100

### Resource availability

#### Lead contact

Further information and requests for resources and reagents should be directed to and will be fulfilled by the lead contact, Dake Qi (dake.qi@umanitoba.ca).

#### Materials availability

The present study did not generate new unique reagents.

### Experimental model and study participant details

#### Human subjects

Human mRNA samples were obtained from a previous overfeeding study to investigate the effects of a positive energy balance on endocrine factors and glucose and lipid metabolism, which has been approved by Newfoundland and Labrador Health Research Ethics Board (HREB).^35^ We also obtained an ethic approval for a secondary use of these samples for the current study (Research portal File# 20200635). All study-related procedures were carried out with written informed consent. The male subjects (Caucasian) including 10 lean (age: 23.3 ± 2.2) and 10 obese (age: 24.3 ± 3.3) were from the city of St. John’s and surrounding area in Newfoundland and Labrador, Canada. All of the subjects agreed to undergo an adipose tissue biopsy for the current mRNA studies. Peripheral venous blood was collected from lean and obese subjects and plasma MIF level was determined with an enzyme-linked immunosorbent assay (ELISA) method according to the protocol from R&D Systems, USA.

#### Mice

All experiments involving mice were conducted in accordance with the Guide for the Care and Use of Laboratory Animals of the National Institutes of Health and were approved by the Internal Animal Committee Review Board of Memorial University of Newfoundland and University of Manitoba. *Par2*^*-/-*^, *Mif* lung Tg and littermate WT male mice with a pure C57BL/6 background (from 20 to 28 weeks of age) were maintained at the Health Science Center Animal Facility in Memorial University of Newfoundland and University of Manitoba, Canada. WT mice (20 weeks) with or without transplantation were housed in individual IVC cages with an artificial 12:12 hr light: dark cycle at room temperature and fed with either normal chow or high palmitic oil diet (41% palmitic oil; #170100, Envigo Teklad Diets) for 8 weeks. Mice were then fasted for six hours, and fasting blood glucose levels were determined from tail venous blood; 2mg/g body glucose or 0.75U/kg insulin then was injected i.p. respectively and blood glucose values were obtained at 0, 15, 30, 60, 90 and 120 min. Serum fatty acid and triglyceride were measured by commercial kits purchased from Wako.

#### Cell culture

3T3-L1 cells isolated from male mouse embryos were cultured in DMEM (11965092, Gibco) with 10% newborn calf serum (16010159, Gibco) and they were differentiated in DMEM containing 10% fetal bovine serum (FBS, 26140079, Gibco), 0.5 mM 3-Isobutyl-1-methylxanthine (IBMX, I7018, Sigma-Aldrich), 0.25 lM dexamethasone (D4902m, Sigma-Aldrich), and 1 μg/ml insulin (I5500, Sigma-Aldrich). Before the experiments, both 3T3-L1 undifferentiated and differentiated cells were briefly serum-starved in DMEM-0.5% newborn calf serum or fetal bovine serum for 8 hours.

### Method details

#### Visceral adipose tissue transplantation

All donor mice (*Par2*^*-/-*^ or WT) at 18 weeks of age were euthanized and their intra-abdominal perigonadal (epididymal) visceral fat depots were isolated and kept in cold saline for the maximum of 30 min until transplantation. Recipient mice (*Par2*^*-/-*^ or WT, 18 weeks) were anesthetized, and their endogenous epididymal fat pads were removed in both sides. The donor fats were then carefully transplanted into the visceral cavity. Recipient mice will be fed with normal chow till 20 weeks of age and then they will receive either normal chow or high fat diet for 8 weeks.

#### Recombinant mouse Pref-1 infusion by mini-pump

WT mice at 20 weeks were initially fed with high palmitic acid diet for 4 weeks. Jugular vein was then cannulated and recombinant mouse Pref-1 (24μg/day/kg) or vehicle was injected via a mini-osmotic pump implanted in a subcutaneous pocket (Alzet model 1004) into the mice accompanied with high palmitic acid diet feeding for the following 4 weeks.

#### Isolation of SVF and adipocyte fractions from adipose tissue

Abdominal fat was excised and digested with 1mg/ml type I collagenase (type I collagenase, LS004196, Worthington Biochemical Company, NJ) for 30min at 37°C. After digestion and centrifugation, the buoyant adipocytes were collected while the cell pellet was retrieved for the stromal vascular fraction (SVF).

#### Flow cytometry

SVF was resuspended in 1% BSA PBS solution and analyzed by flow cytometry. Briefly, SVF was stained with the following antibodies: CD45.2-PerCP (eBioscience; catalog 45-0454-80); F4/80-PE (eBioscience; catalog 12-4801-80); CD11b-APC eFluor 780 (eBioscience; catalog 47-0112-80); CD11c-PE-Cy7 (eBioscience; catalog 25-0114-81); CD206-Alexa Fluor™ 488 (Invitrogen; catalog 53-2061-80); CD301–Alexa Fluor 647 (AbD Serotec; catalog MCA2392A647T); CD31-FITC (Invitrogen; catalog RM5201); CD34-PE (Invitrogen; catalog MA5-17831); anti-Pref-1 antibodies (R&D; catalog MAB8634) and (Invitrogen; catalog MA5-15915); anti-PAR2 antibody (Abcam; catalog 180953); anti-MIF antibody (ab187064; Abcam); anti-Rabbit Alexa Fluor® 488 (Jackson ImmunoResearch; catalog 711-545-152); anti-Rabbit Alexa Fluor™ 647 (Invitrogen; catalog A-21244); anti-Mouse Alexa Fluor® 647 (Jackson ImmunoResearch; catalog 715-605-150). Flow cytometry was performed on a CytoFLEX flow cytometer (Beckman Coulter) and the data were analyzed by using FlowJo v10 software (Becton Dickinson).

#### Expression analysis

Transcript levels for the mouse and human genes of *F2rl1 (PAR2)*, *Mif (MIF)*, *pref-1 (PREF1)*, *PPARγ (PPARG)*, *ATGB1, ATGA5, Tnfa (TNF), Il6 (IL6) and Il1b (IL1B)* (Tables S1 and S2), etc were measured by qPCR. The primer sequences have been listed in Tables S1 and S2. Briefly, RNAs were extracted by TRIzol™ Reagent (Invitrogen) and iScript cDNA Synthesis Kit (Bio-Rad) was used to synthesize complementary DNA. SsoAdvanced™ Universal SYBR® Green Supermix (Bio-Rad) was performed to measure gene expression levels in the QuantStudio 6 Flex System (Applied Biosystems). The delta–delta Ct method was used to quantify gene expressions normalized by *Gapdh*.

Phosphorylation and total levels of ERK, AMPK and ACC, the contents of MIF and p115 in adipose tissue or cells, and the released levels of p115 and MIF in cell culture medium were evaluated by Western blot. Protein extracts were performed in a lysis buffer containing cOmpleteTM, EDTA-free Protease Inhibitor Cocktail (ROCHE). According to the manufacturer’s instructions, protein concentrations were examined using Bio-Rad Protein Assay Dye Reagent (Bio-Rad). Protein quantification was performed by using an iBright™ CL1500 Imaging System (Invitrogen).

#### Immunostaining

Immunohistochemistry staining was performed to identify MIF accumulation in adipose tissue. Immunofluorescence staining was performed on either adipose tissue sections (30μm) or 3T3-L1 cell line cultured on chamber slides. Primary antibodies to the following antigens were used: anti-Pref-1 antibody (R&D; catalog AF8277); PAR2 antibody (Abcam; catalog ab180953); anti-MIF antibody (Torrey Pines Biolabs; catalog TP234) and (R&D; catalog MAB2892); anti-p115 antibody (Proteintech; catalog 13509-1-AP); anti-Pref-1 antibody (Invitrogen; catalog MA5-15915) and anti-ITGB1 (Invitrogen, 14-0292-82). Following the incubation with primary antibodies, the sections were washed three times for 15 min with phosphate-buffered saline and then incubated for 1 hour at room temperature with secondary antibodies. Finally, the images were obtained with an Olympus confocal microscope.

#### PAR2 and AMPK knockdown by siRNA

To temporarily silence PAR2 and AMPK expression in 3T3-L1 undifferentiated cells, 10nM *F2rl1* siRNA (PAR2 siRNA, s65793, ThermoFisher), 30μM AMPK Alpha 1/2 siRNA (sc-45313, Santa Cruz) or non-silencing control siRNA (AM4611, ThermoFisher) was transfected into the cells by using Lipofectamine™ RNAiMAX Transfection Reagent (13778075, Invitrogen) in medium without newborn calf serum and antibiotics as recommended by the manufacturer.

### Quantification and statistical analysis

For the human study, Kolmogorov-Smirnov test was used to examine the data normal distribution. The difference in metabolic indicators and PCR results between lean and obese groups was analyzed using *t*-test. All significant levels were two-tailed tested, and a P value of less than 0.05 was considered as statistically significant.

The food intake, body weight gain, GTT and ITT data in mice were analyzed by multivariate ANOVA. Wherever appropriate, One-way ANOVA with post hoc Tukey’s tests or 2-tailed Student’s *t*-test was used to determine differences between group mean values. The level of statistical significance was set at *P<0.05*.

## Data Availability

• Data reported in this paper will be shared by the lead contact upon request. • This paper does not report original code. • Any additional information required to reanalyze the data reported in this paper is available from the lead contact upon request.
