# Supplementary material for: A pref-1-controlled non-inflammatory mechanism of insulin resistance
Source: iScience. 2023 May 19;26(6):106923. doi: 10.1016/j.isci.2023.106923 (PMC10239698; doi:10.1016/j.isci.2023.106923)
Supplement: Document S1. Figures S1–S16 and Tables S1 and S2 [file mmc1.pdf]

iScience, Volume *26*

Supplemental information

A pref-1-controlled non-inflammatory mechanism of insulin resistance

Yiheng Huang, Donghong Cui, Liujun Chen, Haibin Tong, Hong Wu, Grace K. Muller, Yadan Qi, Shuxia Wang, Jinjie Xu, Xiang Gao, Kathleen E. Fifield, Lingyan Wang, Zhengyuan Xia, Jacqueline L. Vanderluit, Suixin Liu, Lin Leng, Guang Sun, John McGuire, Lawrence H. Young, Richard Bucala, and Dake Qi

Table S1: The description of mouse PCR primer sequences, relate to STAR Methods.

Gene Name	Sequences (5' – 3')
Mif	CGG ACC GGG TCT ACA TAC A<br>TCA AGC GAA GGT GGA ACC GTT
pref-1	CTG TGT CAA TGG AGT CTG CAA G<br>CTA CGA TCT CAC AGA AGT TGC
PPARγ	TGT TAT GGG TGA AAC TCT GGG<br>AGA GCT GAT TCC GAA GTT GG
Fabp4	GTG ATG CCT TTG TGG GAA CCT GGA AG<br>TCA TAA ACT CTT GTG GAA GTC ACG CC
ITGB1	CTG TGG GTG AAT TGT TGC<br>CTA ATC TTT TAA TGT GTC TGT TTG C
ITGA5	TTG CCT GAG TTC CAT CCA AGG<br>CAG AAT CCG GGA GCC TTT GC
F2rl1	AAC ATC ACC ACC TGT CAC GA<br>CAC GTA GGC AGA CGC AGT AA
Tnfa (TNF-α)	CAG GCG GTG CCT ATG TCT C<br>CGA TCA CCC CGA AGT TCA GTA G
Il6 (IL-6)	GAG GAT ACC ACT CCC AAC AGA CC<br>AAG TGC ATC ATC GTT GTT CAT ACA
Il1b (IL-1β)	TGG TGT GTG ACG TTC CCA TT<br>CAG CAC GAG GCT TTT TTG TTG
GAPDH	ATG TGT CCG TCG TGG ATC TGA<br>TGC CTG CTT CAC CAC CTT CTT

Table S2: The description of human PCR primer sequences, relate to STAR Methods.

Gene Name	Sequences (5' – 3')
MIF	CGG ACA GGG TCT ACA TCA A<br>CTT AGG CGA AGG TGG AGT T
PREF1	CTG GAC GGT GGC CTC TAT GAA TG<br>ATC ATC CAC GCA GGT GCC TC
PPARG	ACA GAC AAA TCA CCA TTC GT<br>CTC TTT GCT CTG CTC CTG
TNF (TNF-α)	TGT AGC CCA TGT TGT AGC AAA CC<br>GAG GAC CTG GGA GTA GAT GAG GTA
IL6 (IL-6)	TGG CTG AAA AAG ATG GAT GCT<br>TCT GCA CAG CTC TGG CTT GT
IL1B (IL-1β)	CAG AAG TAC CTG AGC TCG CC<br>AGA TTC GTA GCT GGA TGC CG
β-actin	CCT GTA CGC CAA CAC AGT GC<br>ATA CTC CTG CTT GCT GAT CC

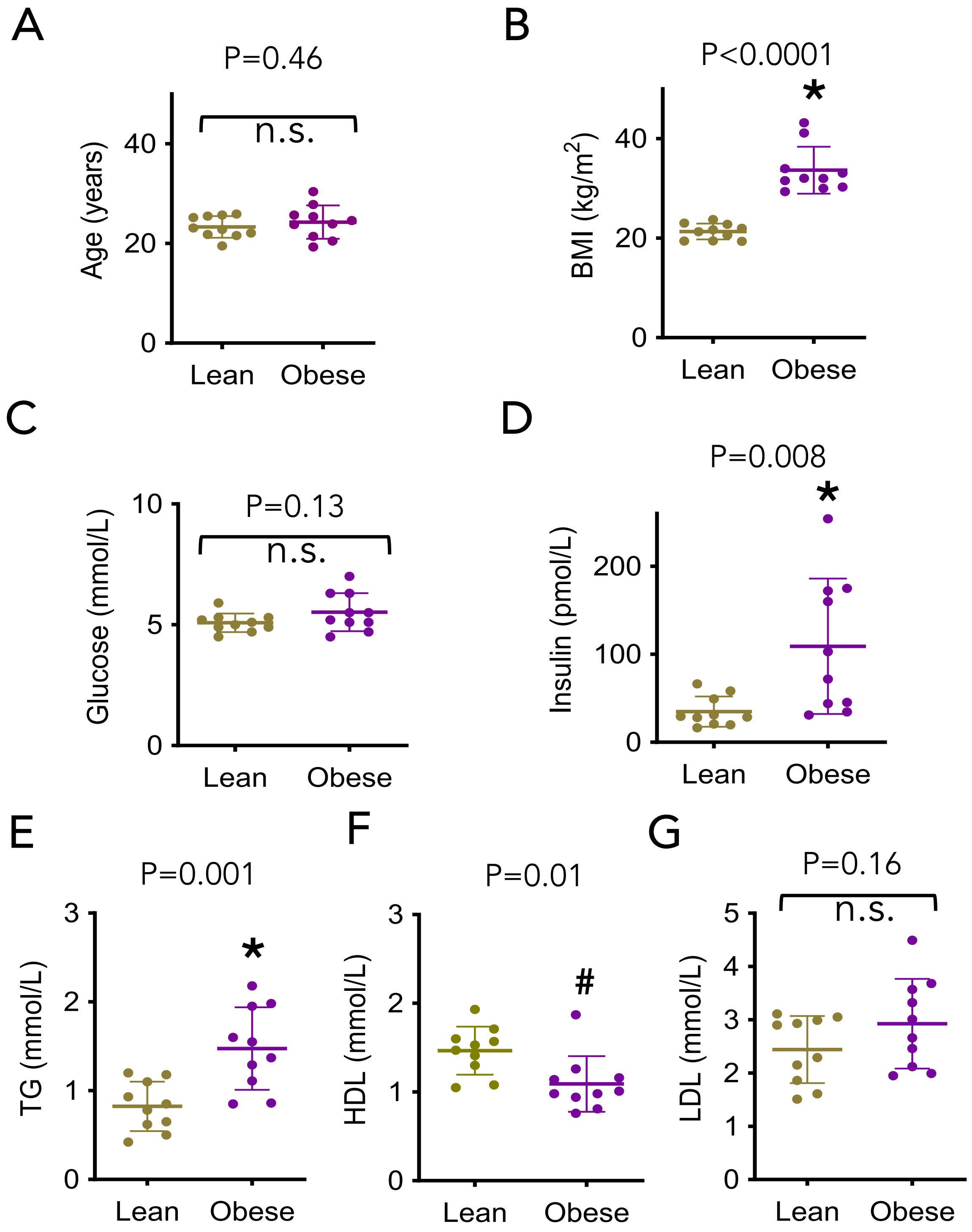

Figure S1: The general characteristics of human subjects, related to Figure 1. 20 male subjects including 10 lean and 10 obese were recruited for the current experiment. Mean ± SD in all the panels; *P ≤ 0.05 increase vs. Lean group; #P ≤ 0.05 reduction vs. Lean group. The n.s. represents no significance.

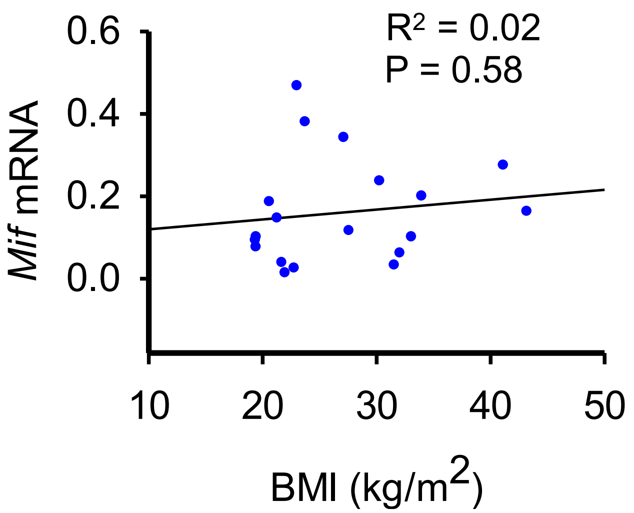

Figure S2: BMI is not correlated with adipose MIF gene expression in human subjects, related to Figure 1.

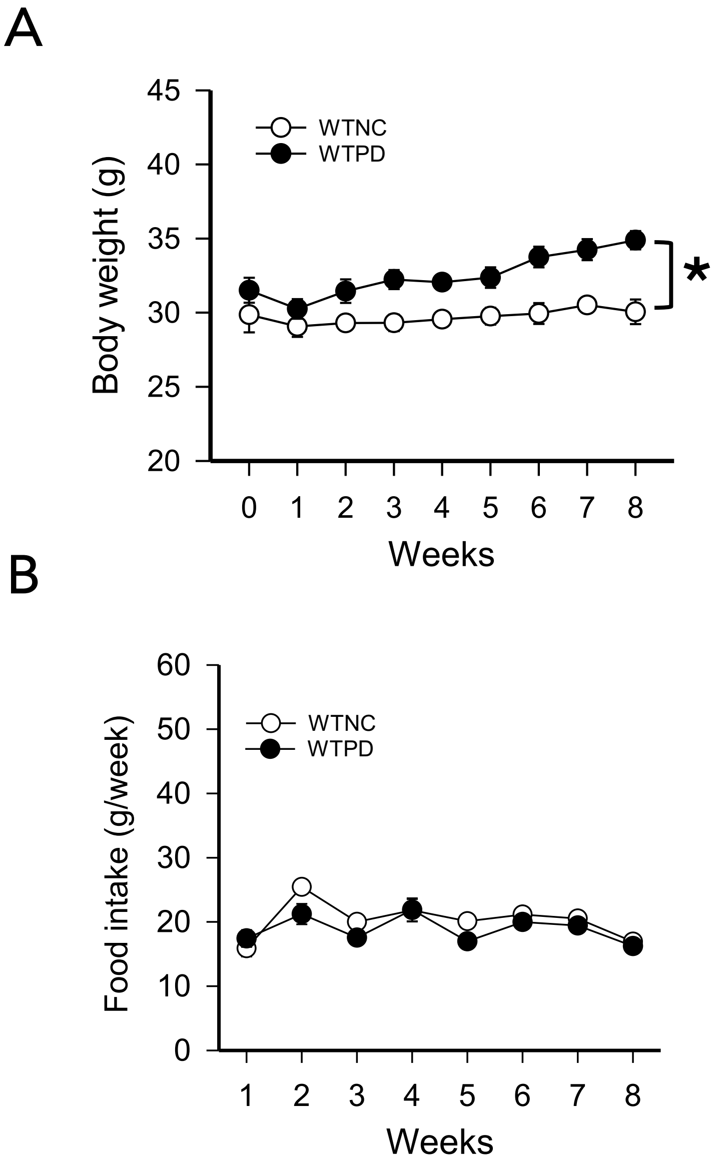

Figure S3: High palmitic acid diet feeding induces obesity in WT mice, related to Figure 1. WT mice at 20 weeks were fed with normal chow (NC) or high palmitic acid (PD) diet for eight weeks. Body weight (A) and food intake (B) was monitored every week. Mean ± SD in all the panels; *P ≤ 0.05 increase vs. NC.

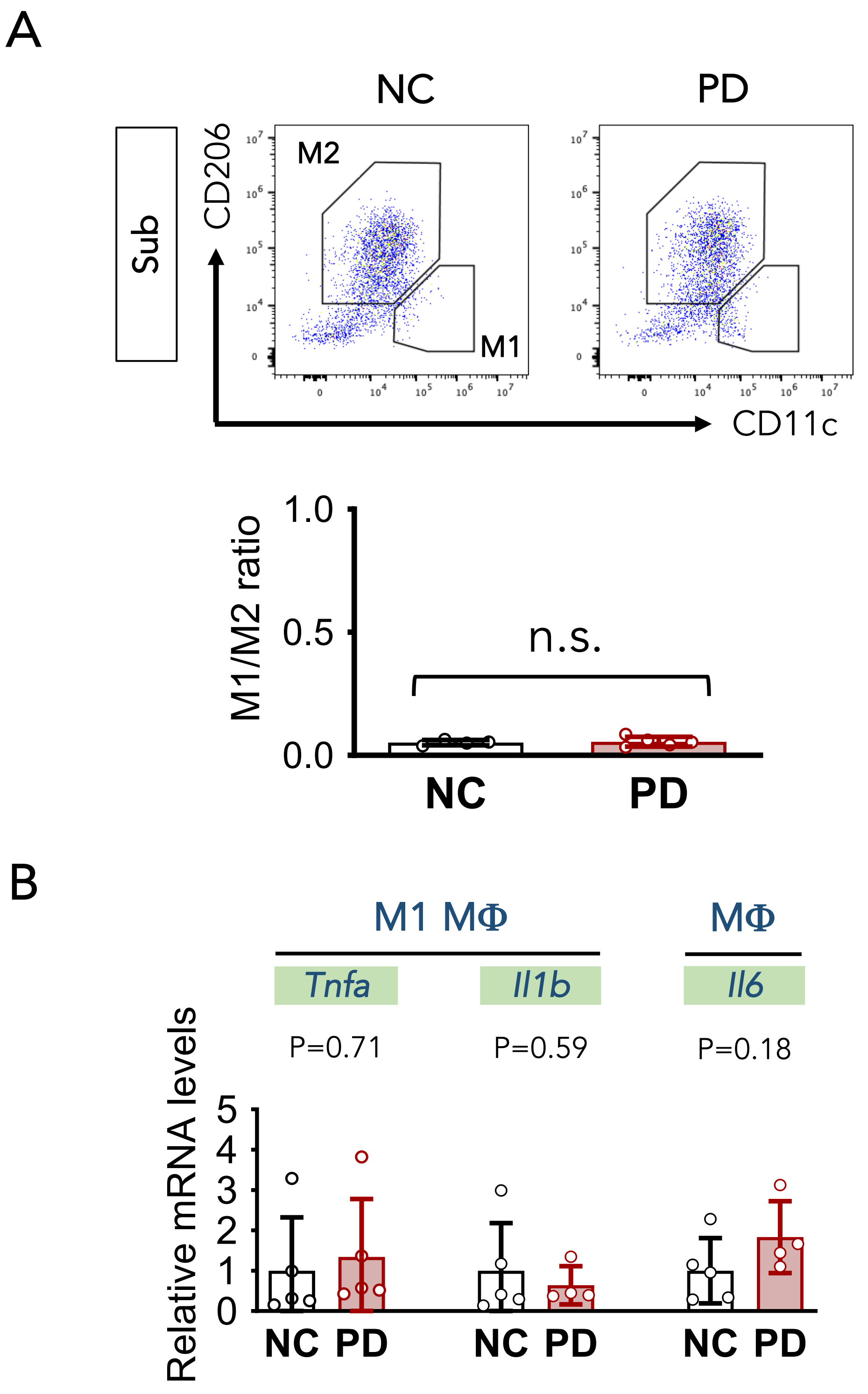

Figure S4. High palmitic acid diet feeding is not associated with inflammation in subcutaneous adipose tissue, related to Figure 1. WT mice at 20 weeks were fed with normal chow (NC) or high palmitic acid (PD) for 8 weeks. M1/M2 ratio (A) and gene expression of *Tnfa*, *Il1b* and *Il6* (B) in femoral adipose tissue were quantified by flow-cytometry and qPCR. Mean ± SD in all the panels; the n.s. represents no significance.

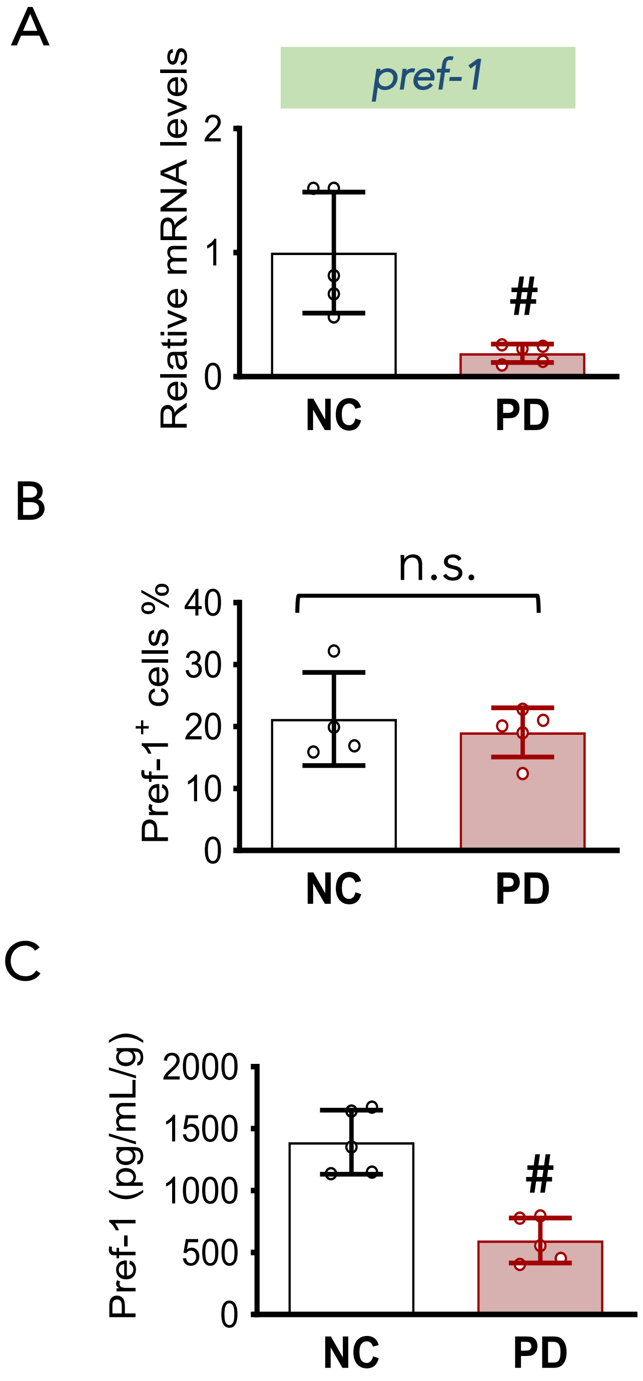

Figure S5: The effects of high palmitic acid diet on Pref-1 expression and release in subcutaneous adipose tissue, related to Figure 1. High palmitic acid diet (PD) reduces pref-1 gene expression (A) without affecting the number of Pref-1+ cells (B) in femoral adipose tissue. PD also decreases Pref-1 release from subcutaneous adipose tissue (C). Mean ± SD in all the panels; #P ≤ 0.05 decrease vs. NC; the n.s. represents no significance.

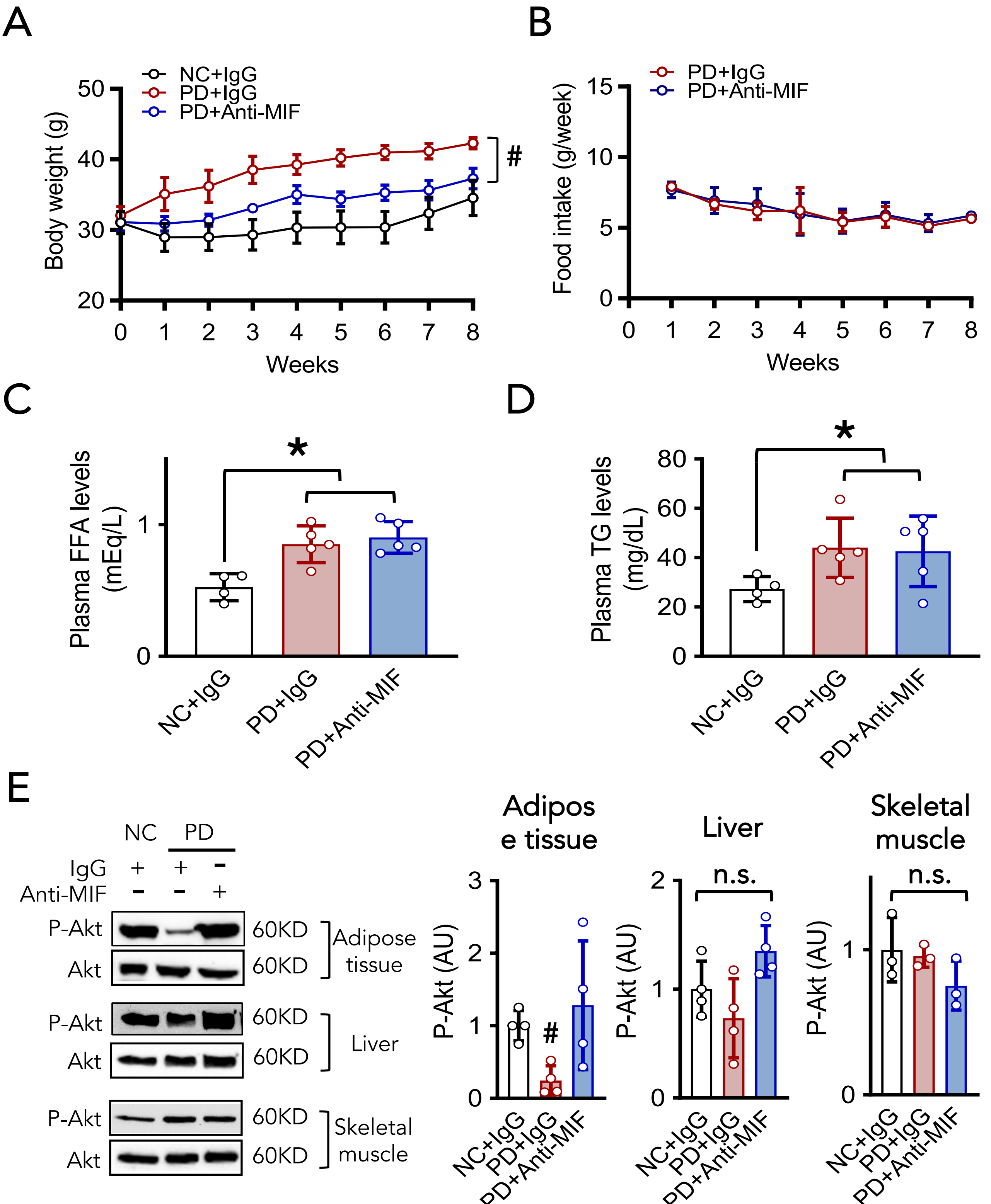

Figure S6: Anti-MIF antibody reverses high palmitic acid diet induced insulin resistance, related to Figure 1. Neutralization of circulating MIF with anti-MIF antibody reduces body weight gain (A) without affecting food intake (B), plasma FA (C) and TG (D) levels following high palmitic acid diet (PD) feeding. The Akt phosphorylation in adipose tissue, liver and skeletal muscle was shown in (E). Mean ± SD in all the panels; #P ≤ 0.05 decrease vs. PD+IgG; *P ≤ 0.05 increase vs. NC+IgG in (C and D), vs other groups in (E); the n.s. represents no significance.

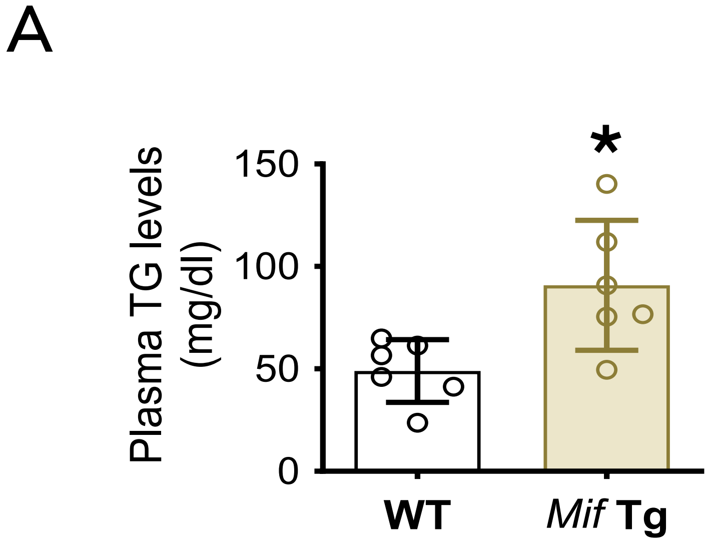

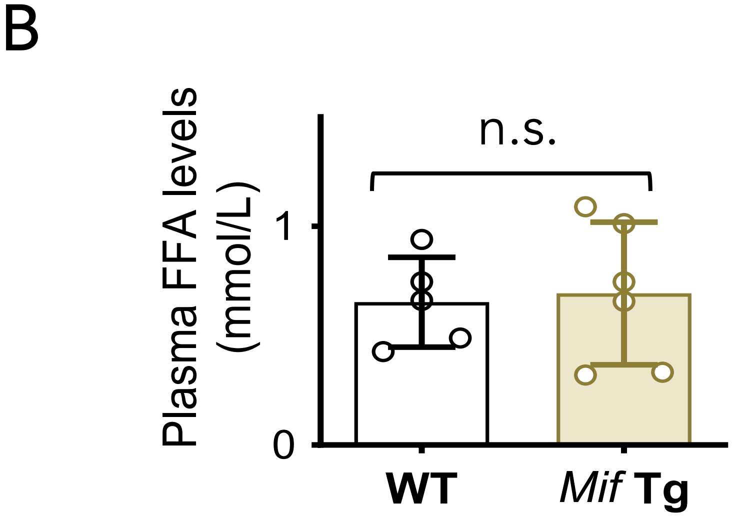

Figure S7. The plasma levels of triglyceride (TG) and free fatty acids (FFA) in WT and Mif lung Tg mice, related to Figure 1. Mean ± SD in all the panels; *P ≤ 0.05 increase vs. WT, the n.s. represents no significance.

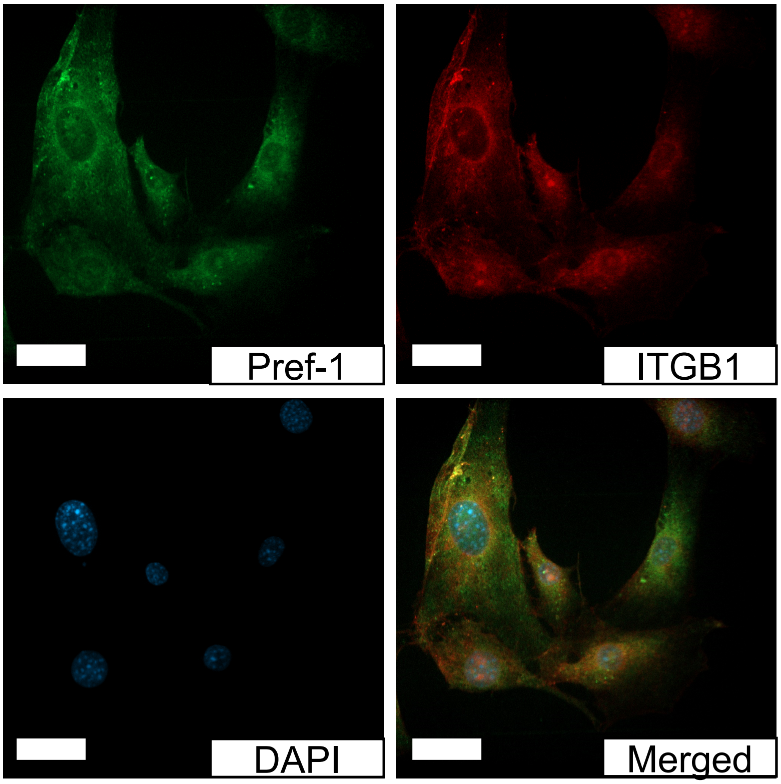

Figure S8: Pref-1 and ITGB1 are colocalized in 3T3-L1 undifferentiated (Pref-1+) cells (Scale bars: 20 μM), related to Figure 3.

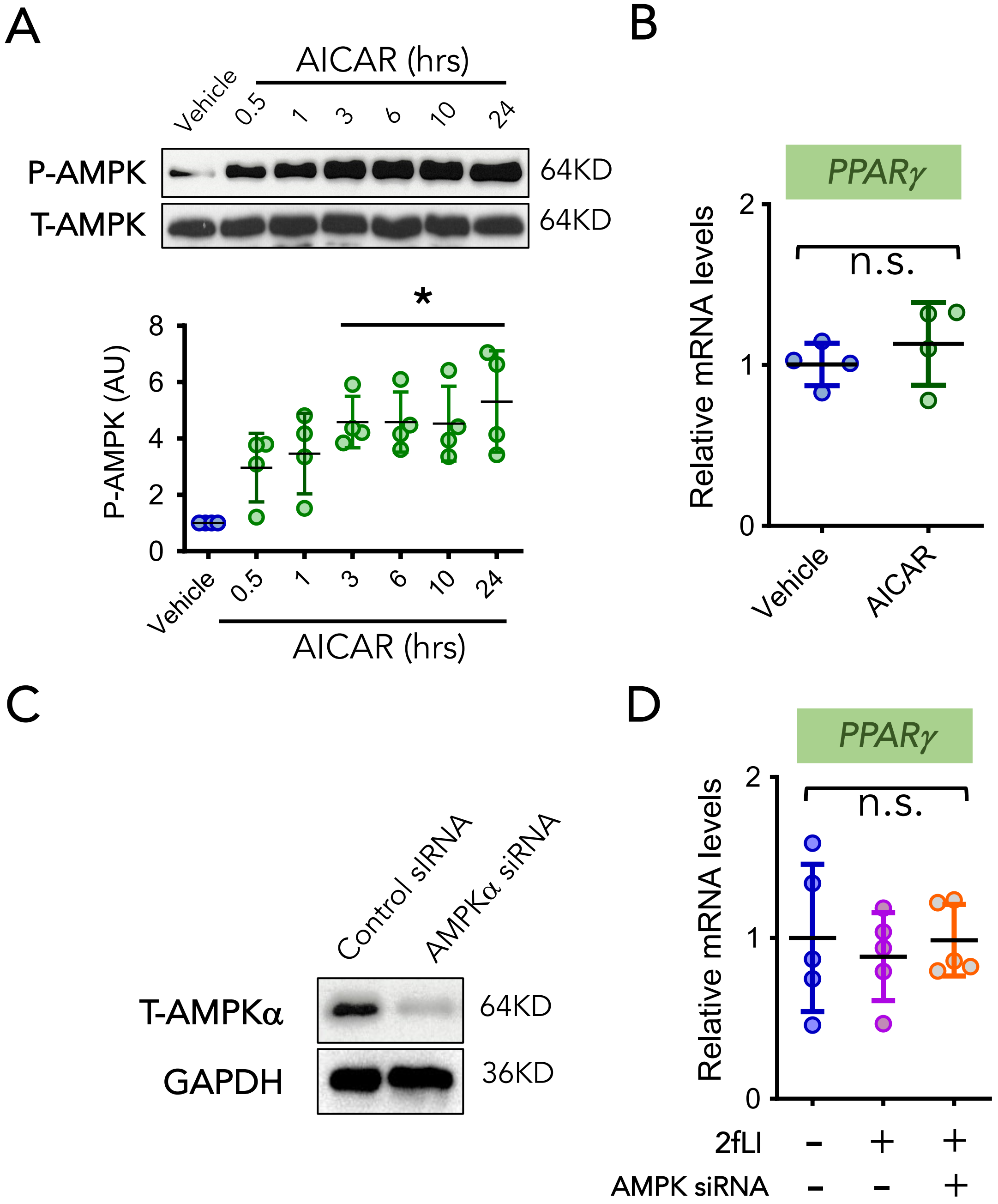

Figure S9: The regulation of AMPK activation does not affect PPARγ gene expression in undifferentiated 3T3-L1 cells, related to Figure 4. The undifferentiated 3T3-L1 cells were incubated with AMPK activator, AICAR (0.25mM) for 0.5 to 24 hours and then AMPK was evaluated by immunoblotting with phosphor- and total AMPKα antibodies (A). (B) *PPARγ* gene expression was evaluated by qPCR. AMPKα was knockdown by AMPKα siRNA (C) in undifferentiated 3T3-L1 cells and *PPARγ* expression (D) was measured with and without 2fLI treatment by qPCR. Mean ± SD in all the panels; *P ≤ 0.05 increase vs. Vehicle; the n.s. represents no significance.

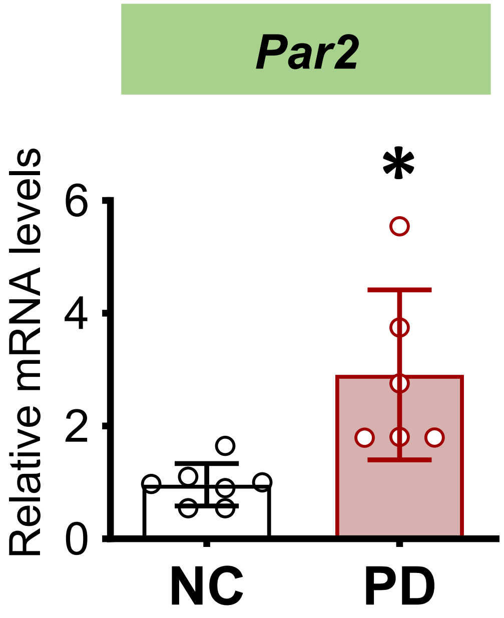

Figure S10: High palmitic acid diet feeding upregulates PAR2 expression in visceral adipose tissue, related to Figure 4. Mean ± SD in the panel; *P ≤ 0.05 increase vs. NC.

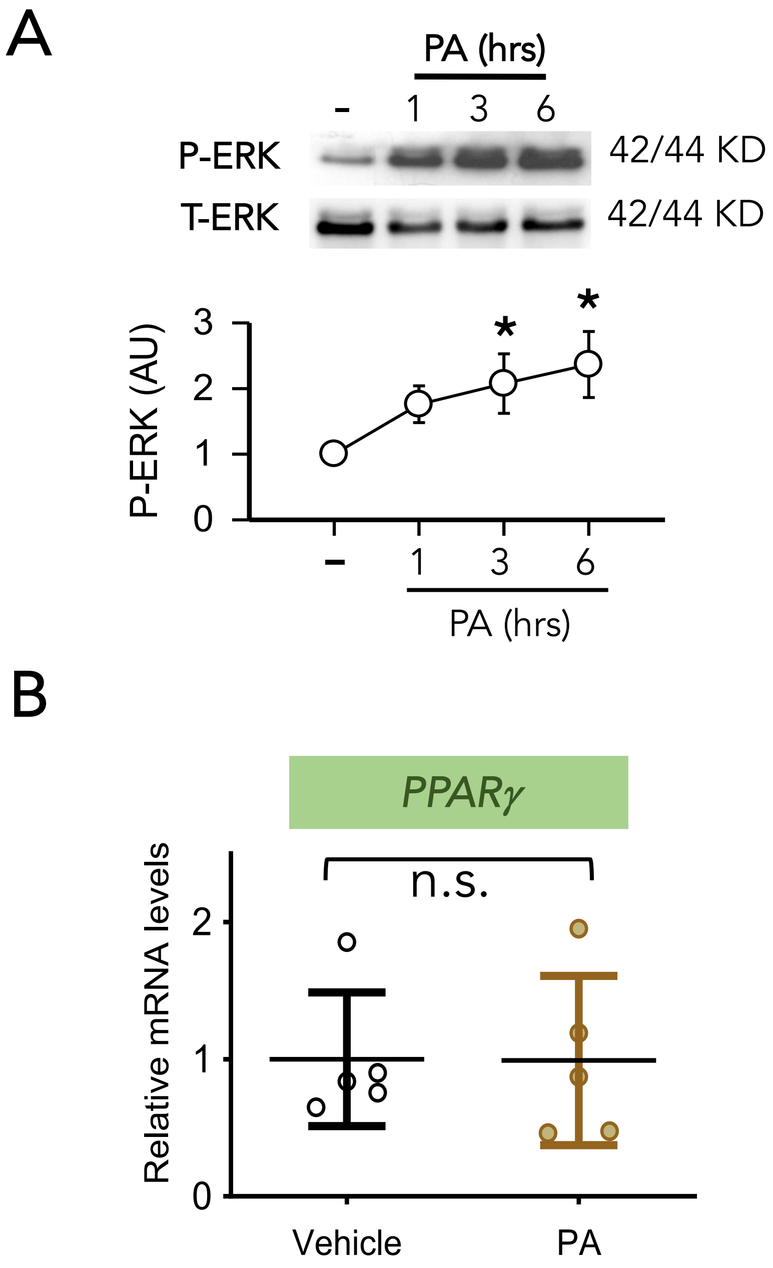

Figure S11: ERK phosphorylation and PPARγ gene expression following high palmitic acid treatment in 3T3-L1 undifferentiated cells, related to Figure 4. The undifferentiated 3T3-L1 cells (Preadipocytes) were incubated with or without high palmitic acid (200μM) from 1 to 6 hours and then the isolated protein was immunoblotted with phosphorylated ERK and total ERK antibodies (n=6 each group) (A). *PPARγ* gene expression (B) was evaluated by qPCR. Mean ± SD in all the panels; *P ≤ 0.05 increase vs. Vehicle, the n.s. represents no significance.

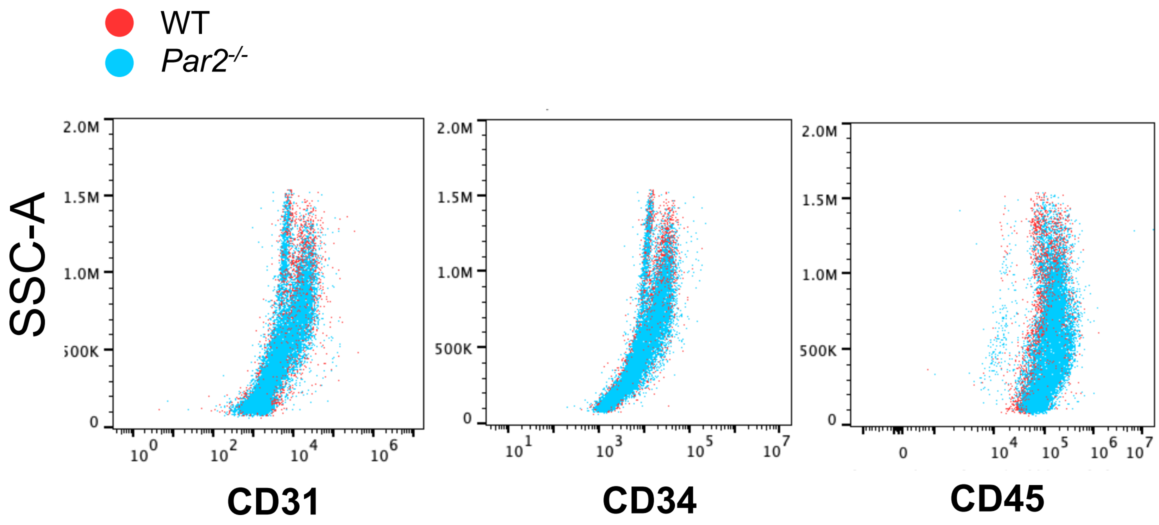

Figure S12: Cells with CD31+, CD34+ or CD45+ in Pref-1+ cells isolated from visceral adipose tissue in 25-week WT and *PAR2*$^{-/-}$ mice by flow cytometry, related to Figure 5.

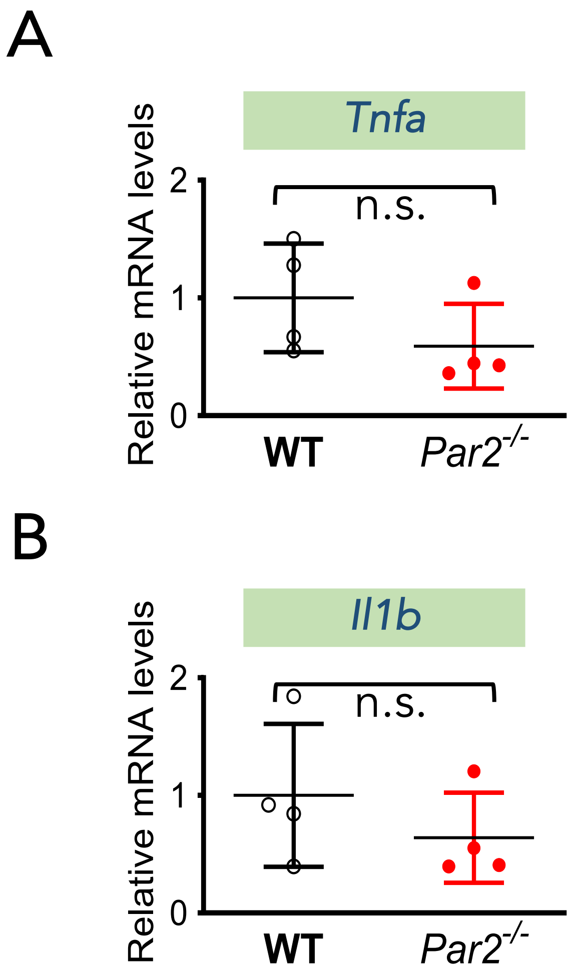

Figure S13: The gene expression of inflammatory factors in visceral adipose tissues from 25-week WT and Par2$^{-/-}$ mice, related to Figure 5. *Tnfa* (A) *and Il1b* (B) gene expression was evaluated by qPCR. Mean ± SD in all the panels; the n.s. represents no significance.

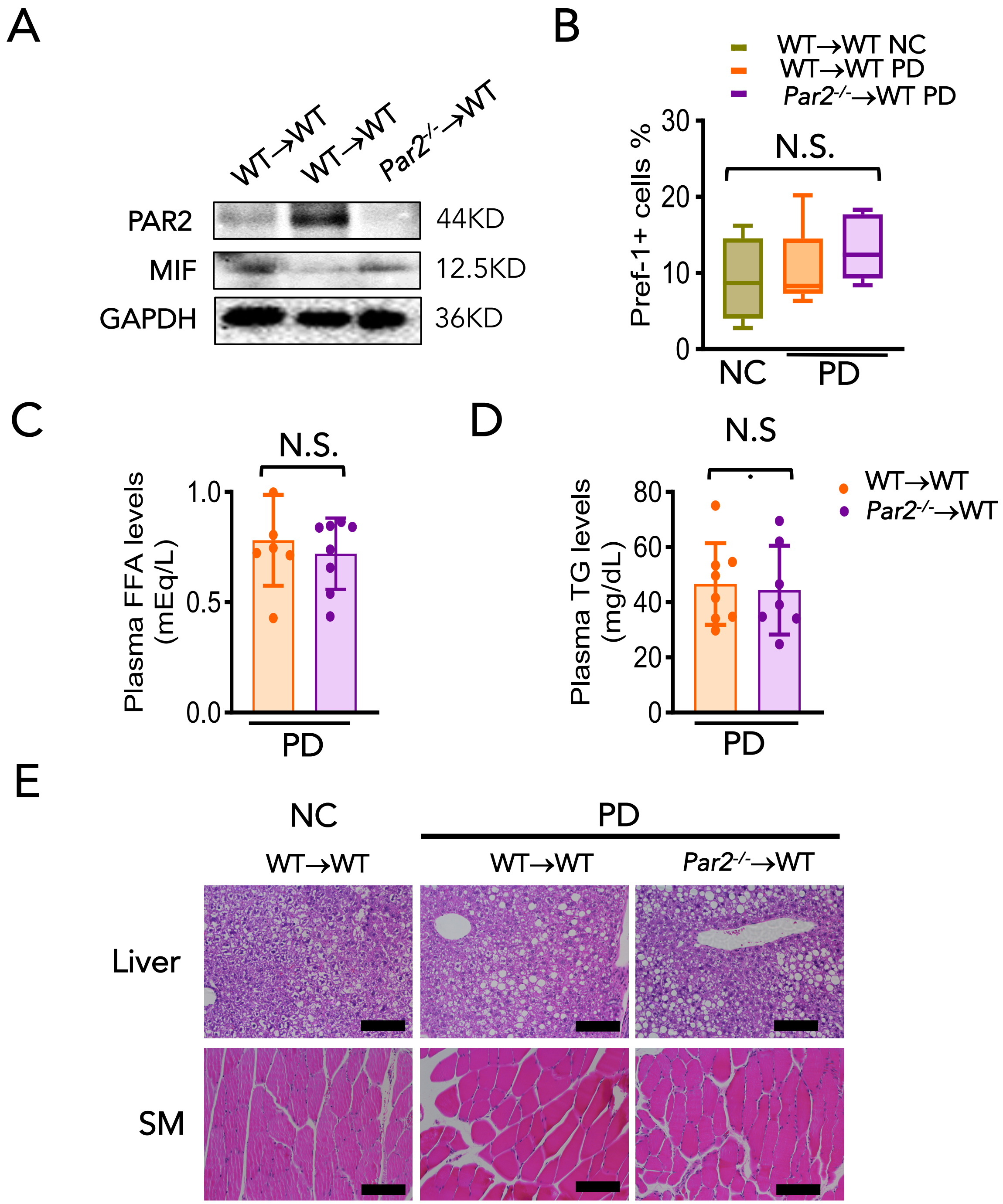

Figure S14: The metabolic characteristics of WT mice transplanted with Par2$^{-/-}$ visceral adipose tissue following high fat diet feeding, related to Figure 6. WT mice at 18 weeks were anesthetized and their visceral adipose tissues (epididymal) were removed. Visceral adipose tissues (epididymal) from WT or *Par2$^{-/-}$* donors were transplanted into these WT mice. Following 2-week recovery, these mice at 20 weeks were fed with normal chow (NC) or high palmitic acid diet (PD) for 8 weeks. PAR2 and MIF protein expression was evaluated in the transplanted adipose tissues by western blot (A). The components of pref-1+ cells were quantified by flow-cytometry (B). The levels of plasma fatty acid (FA) (C) and triglyceride (TG) (D) were also quantified and lipid storage in liver and skeletal muscle (SM) (E) was identified by HE staining. Mean ± SD in all the panels; the n.s. represents no significance.

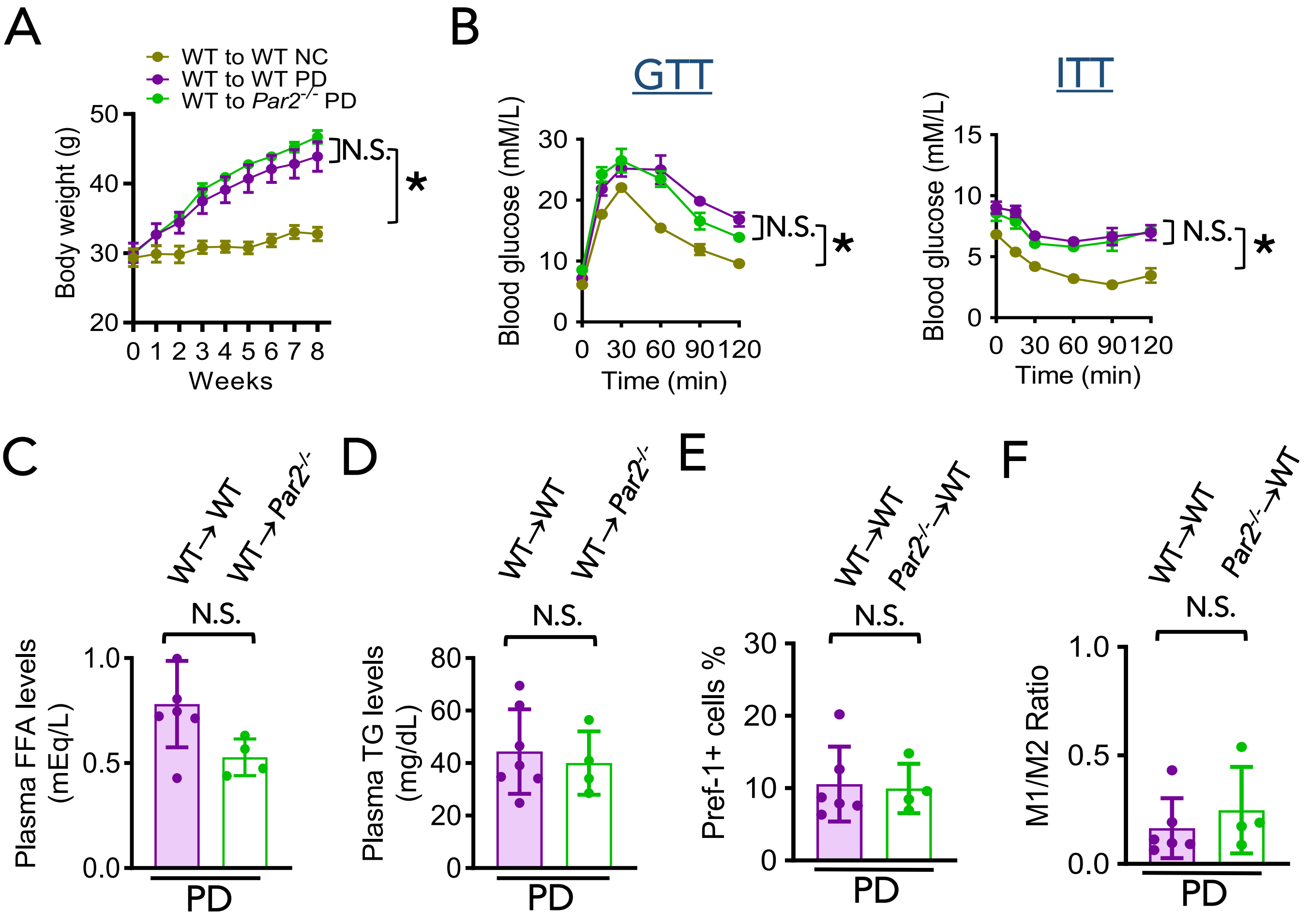

Figure S15: The metabolic characteristics of Par2$^{-/-}$ mice transplanted with WT adipose tissue following high fat diet feeding, related to Figure 6. WT and *Par2*$^{-/-}$ mice at 18 weeks were anesthetized and transplanted with WT visceral adipose tissue (epididymal). Following 2-week recovery, these mice at 20 weeks were fed with normal chow (NC) or high palmitic acid diet (PD) for 8 weeks. Body weight gain was monitored in (A) and insulin resistance was quantified by ip glucose tolerance and insulin tolerance tests (B). Plasma fatty acid (FA) and triglyceride (TG) levels were measured in (C) and (D). The components of adipose pref-1+ cells and M1/M2 ratio were evaluated by flow-cytometry (E and F). Mean ± SD in all the panels; *P ≤ 0.05 increase vs WT to WT NC, the n.s. represents no significance.

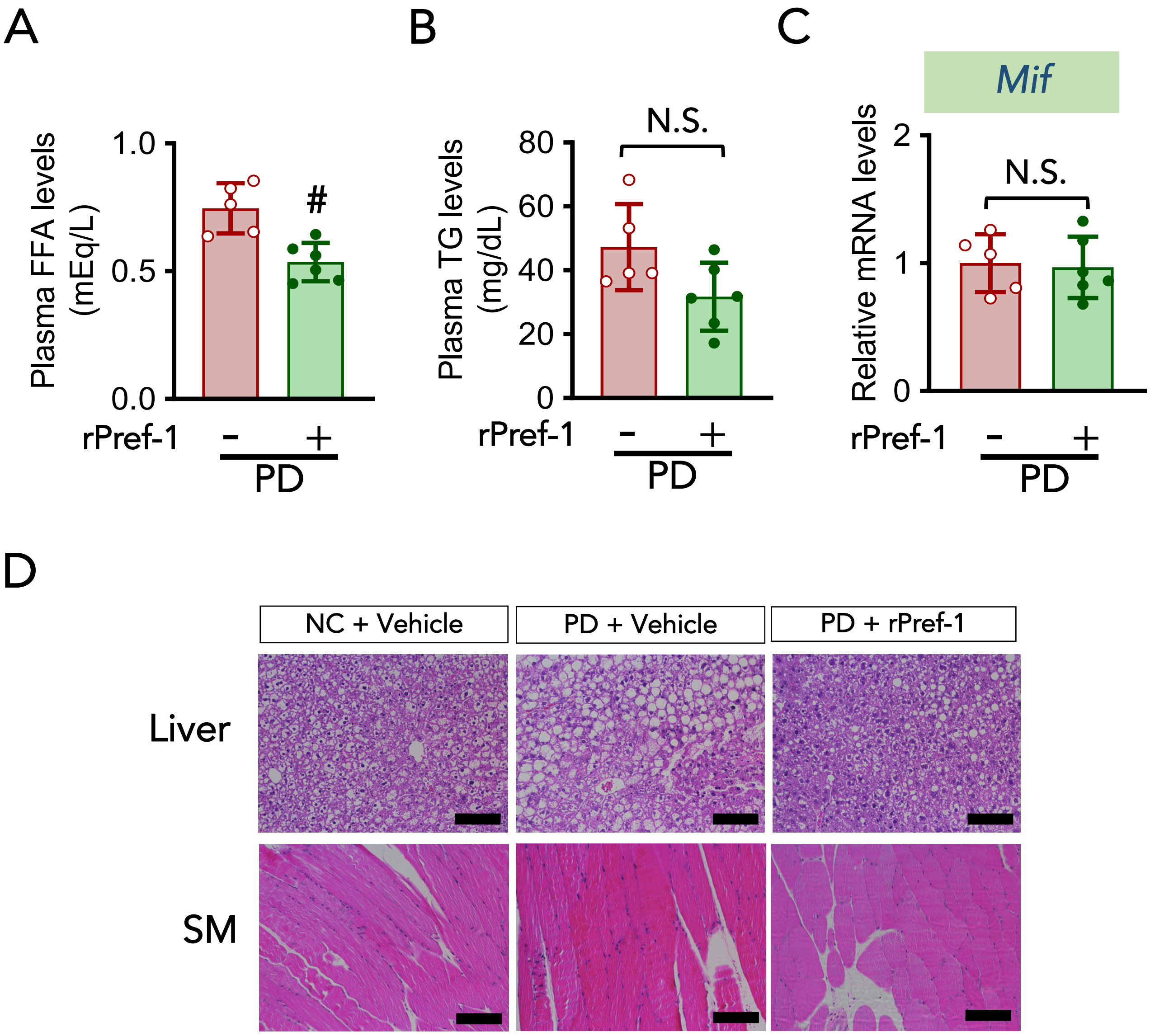

Figure S16: The effects of pref-1 infusion on high palmitic acid diet induced metabolic dysfunction, related to Figure 6. WT mice at 20 weeks were fed with NC or PD for 8 weeks. During the last 4 weeks, the mice were infused with vehicle or recombinant pref-1 protein by osmotic pump. Plasma levels of fatty acid (FA) and triglyceride (TG) were measured in (A) and (B). *Mif* gene expression was quantified by qPCR (C) and lipid storage in liver and skeletal muscle (SK) were evaluated by HE staining in (D, Scale bars: 100 μM). Mean ± SD in all the panels; #P ≤ 0.05 reduction vs vehicle, the n.s. represents no significance.
